# Impact of a Single Nucleotide Change or Non-Nucleoside Modifications in G-Rich Region on the Quadruplex–Duplex Hybrid Formation

**DOI:** 10.3390/biom11081236

**Published:** 2021-08-18

**Authors:** Dorota Gudanis, Karolina Zielińska, Daniel Baranowski, Ryszard Kierzek, Piotr Kozłowski, Zofia Gdaniec

**Affiliations:** Institute of Bioorganic Chemistry, Polish Academy of Sciences, 61-704 Poznan, Poland; kczajczynska@ibch.poznan.pl (K.Z.); danibari@ibch.poznan.pl (D.B.); rkierzek@ibch.poznan.pl (R.K.); kozlowp@ibch.poznan.pl (P.K.)

**Keywords:** quadruplex–duplex hybrid, RNA G-quadruplexes, non-canonical nucleic acid structures, non-nucleotide chemical modifications, abasic, aliphatic linkers, o-BMVC G-quadruplexes ligand, single nucleotide change

## Abstract

In this paper, a method to discriminate between two target RNA sequences that differ by one nucleotide only is presented. The method relies on the formation of alternative structures, i.e., quadruplex–duplex hybrid (QDH) and duplex with dangling ends (Dss), after hybridization of DNA or RNA G-rich oligonucleotides with target sequences containing 5′–GGGCUGG–3′ or 5′–GGGCGGG–3′ fragments. Using biophysical methods, we studied the effect of oligonucleotide types (DNA, RNA), non-nucleotide modifications (aliphatic linkers or abasic), and covalently attached G4 ligand on the ability of G-rich oligonucleotides to assemble a G-quadruplex motif. We demonstrated that all examined non-nucleotide modifications could mimic the external loops in the G-quadruplex domain of QDH structures without affecting their stability. Additionally, some modifications, in particular the presence of two abasic residues in the G-rich oligonucleotide, can induce the formation of non-canonical QDH instead of the Dss structure upon hybridization to a target sequence containing the GGGCUGG motif. Our results offer new insight into the sequential requirements for the formation of G-quadruplexes and provide important data on the effects of non-nucleotide modifications on G-quadruplex formation.

## 1. Introduction

In addition to the well-known double helix, nucleic acids can form a variety of different conformations. G-quadruplexes (G4) are structures that have received significant attention in recent years. The canonical, unimolecular G4 structures are formed from DNA or RNA G-quadruplex forming sequences (G4FS) comprising four G-tracts, and they fold into stacks of at least two guanine tetrads stabilized by Hoogsteen hydrogen bonds. Four G-tracts are necessary to form a G-quadruplex; however, they can also be part of two (bimolecular G4) or four separate strands (tetramolecular G4) [1]. The folding of G-quadruplexes into parallel, antiparallel, or hybrid-type topologies and their stabilities depends on the intramolecular factors, including sequence (number of G residues in G-tracts, the sequence of the loops, 5′/3′-flanking sequences) [2], environmental conditions (type of cations, pH, co-solvent) [3], and presence of small molecules (G4-stabilizing or G4-destabilizing ligands) [4] or proteins [5,6]. Polymorphism is inherent in G4 formation for most DNA G4-forming sequences. In contrast, RNA G-quadruplexes have long been considered as structurally monomorphic. However, RNA G4s with unusual motifs and arrangements of G-tetrads have been reported recently [7,8]. For example, in G4 cores of Spinach and Mango aptamers, some guanosine residues assume the *syn* conformation, consequently inducing the unusual antiparallel topology of G4 RNA [9,10,11]. Many modified nucleotides or non-nucleotide linkers have been used to manipulate G-quadruplex structures [12,13,14,15,16,17,18]. Rational incorporation of these modifications into G4FS can be used to stabilize particular conformers. Although the presence of G-tetrads is required to form G-quadruplexes, non-canonical tetrads have also been observed in high-resolution structures or models of G4s [8,19,20,21,22,23,24,25,26,27]. G4s are more tolerant to mutations than was previously thought. For example, it was found that G4 structures with G-tetrads replaced by non-guanosine tetrads can retain their biological functions [28]. Environmental conditions are one of the factors that can trigger the formation of unusual tetrads. For example, molecular crowding can destabilize the hairpin structure, thus promoting the formation of a G-quadruplex containing a GGUU tetrad [29]. It was also observed that intracellular mRNA can be entrapped in the formation of kinetically favored metastable hairpin-like structures that disturb the formation of the thermodynamically favored G-quadruplex [30]. At present, it is increasingly clear that the principles of predicting G4FS are more complicated than originally thought. Recently, the G4-seq technique, developed to detect G4 structures (next-generation sequencing, NGS), was used to identify over 700,000 DNA G4-forming sequences in the human genome [31]. This G4 mapping doubled the number of DNA G-quadruplexes predicted in silico by standard algorithms [32]. Using a similar rG4-seq approach, more than 13,000 G4FS were identified in 3000 human mRNAs [33]. In addition to mRNA, RNA G4 may also be present in mitochondrial RNA, tiRNA, piRNA, lncRNA, and miRNA [34,35,36,37,38]. Taking into account that these newly identified G4-forming sequences have the potential to fold into an unconventional class of G4 structures containing two-layer G-tetrads, bulges, or longer loops, the known repertoire of G4 structures may still be incomplete [31,33]. The most recent research has revealed that G-tracts involved in G-quadruplex formation can be located as far as 7–20 nucleotides away in the human genome [39]. The long loop sequences may adopt stem-loop secondary structures, and their formation was reported to accelerate DNA G4 folding [40]. G-quadruplexes containing stem-loop motifs are called quadruplex–duplex hybrid structures (QDHs). Structural studies highlighted a large diversity across their conformations [41,42,43,44,45,46,47]. The same G-rich sequence can adopt more than one hybrid form. For example, the coexistence of two different structures of DNA QDHs was observed for fragments of PIM1 or EGFR oncogenes [48,49]. Two separate oligonucleotides can assemble into bimolecular QDHs involving DNA-DNA, RNA-RNA, or DNA-RNA strands [50,51,52,53,54]. The endogenous QDH structures have been proposed as an attractive target for the regulation of oncogene expression in cells. When designing new small-molecule drugs, differences in QDH structure generating different potential sites for interaction with G4 stabilizing ligands should be taken into account [54,55,56]. For example, for quadruplex–duplex hybrid structures, an interesting approach based on the simultaneous binding of two ligands connected by a linker, PIP and cIKP, with different affinities for the duplex and G-quadruplex, has been proposed [55].

Previous reports suggested that most RNA G-quadruplexes exist in living cells in an unfolded state; however, recent studies proposed a model of a dynamic G4 RNA folding equilibrium controlled mainly by ions and G-quadruplex binding proteins [5,57,58,59]. The visualization of G4 RNA using the QUMA-1 ligand that binds only to the existing G4 structure confirmed a dynamic equilibrium between G4 RNA folded and unfolded states [60]. In general, the number of G4 RNA structures detected in cells depends on the technique used [33,60]. Nevertheless, detection of G4 RNA with G4-specific antibodies [61], G4 ligands [57,60], and a G-quadruplex-triggered fluorogenic hybridization probe [62,63] unequivocally confirmed their presence, both in vitro and in cells. Currently, G4 RNAs are important objects of research in biology, with a particular emphasis on the role of G4 in the flow of genetic information in cells [30] and G4-associated diseases [36,64,65,66,67,68,69]. Recently, we have demonstrated the sequence-specific targeting of a 56 nt long EGFR mRNA fragment comprising two distant GGGG tracts by RNA oligonucleotides composed of a chemically modified G-rich segment and a flanking 16 nt fragment complementary to mRNA EGFR [52]. As a consequence, the formation of a bimolecular RNA quadruplex–duplex hybrid structure containing a 28 nt long external loop adopting two duplex-stem structures was observed in vitro and in cells. We also noted the possibility of using RNA G-rich oligonucleotides conjugated to a fluorescent probe in a visualization of the density of the endogenous EGFR mRNA in MCF-7, HeLa, and A431 cancer cells. 

**Figure 1:**
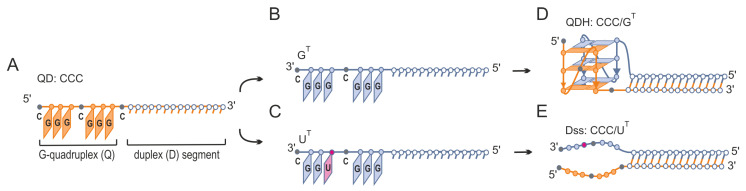
Schematic presentation of the recognition of G^T^ and U^T^ targets by the CCC oligonucleotide. CCC oligonucleotide (**A**) and target sequence with two GGG-tracts, G^T^ (**B**) forms a quadruplex–duplex structural hybrid (QDH) (**D**). CCC oligonucleotide hybridized to the U^T^ target (**C**) forms a duplex with long dangling ends (Dss) (**E**).

Here, we demonstrate that target G-rich sequences that differ by a single nucleotide (5′–GGGC**U**GG–3′ vs. 5′–GGGC**G**GG–3′) can be targeted by G-rich oligonucleotides (QD) in a structure-specific manner. The change in a single nucleotide from uridine to guanosine results in the formation of a second 3 nt GGG tract in a target sequence and triggers the formation of a G-quadruplex motif (Figure 1). Using biophysical methods, we demonstrated the formation of two alternative structures, quadruplex–duplex hybrid (QDH) and duplex with dangling ends (Dss), depending on the target sequence (Figure 1). We undertook systematic studies on the effects of the type of oligonucleotide (DNA, RNA), non-nucleotide modifications (aliphatic linkers or abasic), and the covalently attached G4 ligand on the ability of G-rich oligonucleotides to recognize target molecules and to fold into QDH and Dss structures. Additionally, we proved that the replacement of a single nucleotide loop or junction with a non-nucleotide modification led to the formation of the QDH structures without affecting their stabilities. We also found that the presence of chemical modifications can induce the formation of the G4 motif on the U^T^ target sequence despite the lack of one of the guanosine residues in the G-tract. The presented results may have implications for the structure-based design of G-rich antisense oligonucleotides and enable a more rational design of G-rich oligonucleotides in anticancer therapy.

## 2. Materials and Methods

All RNA and DNA sequences used in the study are shown in Table 1.

### 2.1. Synthesis and Purification of DNA and RNA Oligonucleotides

Synthesis of DMT-ON DNA and RNA oligonucleotides at the 1.0 µmole scale was performed according to routine procedures on a MerMade12 (BioAutomation, Irving, TX, USA) or a Gene World DNA synthesizer (K&A, Schaafhein, Germany) under the conditions recommended by the manufacturer. Samples were cleaved from the solid support, deprotected using standard procedures (Glen Research, Glen-Pak RNA, or DNA Cartridge Purification (DMT-ON), Sterling, VA, USA). The only exception was MMTr-C6-amino oligoribonucleotide (NH_2-_CCC), which was removed from the solid support and deprotected by overnight treatment with concentrated ammonia and ethanol (3:1) at 55 °C. Next, the solvent was evaporated to dryness, and the 2′-silyl protection was removed by treatment with 1.0 M triethylammonium fluoride at 65 °C for 2.5 h. In the next step, the precipitation was made by the addition of 5 mL of 1-butanol, and the samples were stored at −20 °C for 1 h. The precipitate was separated from the solution by spinning at 5000 rpm, 4 °C, for 10 min. The MMTr group was removed using 80% acetic acid/water for 30 min, and the solvent was evaporated to dryness. The NH_2_-CCC oligonucleotide was precipitated in the presence of 2% NaClO_4_/acetone. All oligonucleotides (Table 1) were then purified by RP-HPLC on a 1260 Infinity HPLC system (Agilent Technologies, Santa Clara, CA, USA) using XTERRA 5 µm, C18, 150 × 4.6 mm column with buffer A (0.1 M NH_4_HCO_3_/H_2_O) and buffer B (100% CH_3_CN) at a 1.5 mL/min flow rate, 70 °C. The buffer gradient was as follows: (1) 0–2 min 0% B; (2) 2–10 min 0–10% B; (3) 10–12 min 10–50% B; (4) 12–13 min 50–0% B; and (5) 13–30 min 0% B. UV detection was performed at λmax 268 nm. 

All oligonucleotides were desalted using Amicon^®^ Ultra 3K centrifugal filters (Merck, Millipore, Darmstadt, Germany) by loading on the filter, washing several times with 4000 µL MilliQ water, and then washing successively against ∼150 mM LiCl and against water. 

To prevent dimerization of G-quadruplexes, experiments were performed in the presence of 50 mM KCl instead of 150 mM KCl [22,52]. 

### 2.2. NH_2_-CCC Labeling with o-BMVC-C3

o-BMVC-C3-NHS was coupled to the NH_2_-CCC according to a published procedure [52]. The purity and homogeneity of the oligo-o-BMVC-C3 conjugates were verified by 15% denaturing gel electrophoresis (Figure S1).

### 2.3. Denaturing Electrophoresis of RNA Oligo-o-BMVC-C3

First, 350 pmole RNA oligo-o-BMVC-C3 was suspended in 4 µL water and 4 µL of 8 M urea (in 8 µL final volume). Next, the sample was heated at 95 °C for 4 min, cooled to room temperature, and loaded on 15% TBE-Urea gels (Invitrogen, Thermo Fisher Scientific, Pittsburgh, PA, USA). Denaturing gel electrophoresis experiments were performed in 1.5 × TBE buffer. The electrophoresis experiment was run at 180 V for 2 h, and the gel was visualized by UV shadowing (Figure S1).

### 2.4. Non-Denaturing Electrophoresis of Oligonucleotides

First, 350 pmole of oligonucleotides were suspended in 50 mM KCl, 10 mM phosphate potassium buffer, pH 6.8 or 150 mM NaCl, 10 mM phosphate sodium buffer, pH 6.8 (in 6 µL final volume). Next, samples were heated at 95 °C for 3 min and gradually cooled to room temperature. Subsequently, samples were mixed with 2 µL of 50% glycerol and loaded on 20% TBE gels (Invitrogen, Thermo Fisher Scientific, Pittsburgh, PA, USA). O’RangeRuler 5 bp and DNA ladder containing 50 and 100 bp as brighter bands (Thermo Fisher Scientific, Pittsburgh, PA, USA) were used as molecular markers. Gel electrophoresis experiments performed in a 0.5 × TBE buffer were run at 160 V for ~2 h at 4 °C (an ice bath), and the gels were first visualized by UV shadow (image) and then stained by N-methyl mesoporphyrin IX (NMM; Frontier Scientific, Newark, DE, USA). After post-staining the gels with NMM, the gels were scanned with a Fuji FLA-5100 imaging system (Fujifilm Life Sciences, Cambridge, MA, USA). 

The last gel was viewed by UV shadow to visualize all RNA (image) and exposed to 473 nm light to visualize fluorescently labeled G-quadruplex (o-BMVC-C3-CCC/G^T^ hybrid). 

### 2.5. NMR Experiments

NMR experiments were performed on a 700 MHz Bruker AVANCE III spectrometer (Bruker Corporation, Billerica, MA, USA) equipped with a QCI CryoProbe. The oligonucleotides were annealed by heating to 90 °C and then slowly cooled to room temperature. The ^1^H NMR spectra of oligonucleotides were recorded at 25 °C or in the 25–75 °C range in 3 mm thin wall tubes with a sample volume of 200 μL. The final concentration of QD/G^T^, QD/U^T^ was ~0.1 mM in a buffer containing 50 mM KCl, 10 mM phosphate potassium, or 150 mM NaCl, 10 mM phosphate sodium at pH 6.8. A mixture of 90% H_2_O and 10% D_2_O was used for experiments undertaken to study imino protons. The water signal was suppressed by excitation sculpting with a gradient pulse. Spectra were processed and prepared with TopSpin 3.2 Bruker Software.

### 2.6. UV Thermal Denaturation Curves

First, 590 pmole of QD/G^T^ and QD/U^T^ oligonucleotides were dissolved in a buffer containing 50 mM KCl, 10 mM phosphate potassium buffer, or 150 mM NaCl, 10 mM phosphate sodium buffer at pH 6.8. Thermal denaturation curves were obtained by monitoring at 260 and 295 nm with a JASCO V-650 spectrophotometer (JASCO International Co., Ltd., Tokyo, Japan) using quartz optical cuvettes of 0.5 path length with the sample volume of 150 µL. Samples were protected against evaporation by silicone oil. Before measurements, the cuvettes filled with samples were spun at 5000 revolutions per minute for 3 min to avoid the formation of air bubbles during measurements. The temperature range was 20–90 °C, using a scan rate of 0.5 °C min^−1^. Spectra were processed and prepared using Origin 8 Software (OriginLab Corporation, Northampton, MA, USA). The melting temperature was determined by the local maximum of the first derivatives of the absorbance vs. temperature curve.

### 2.7. Fluorescence Measurements

One equivalent of NMM was mixed with QD/G^T^ or QD/U^T^ (25 µmol/L) in a buffer containing 10 mM potassium phosphate, 50 mM KCl, 6.8 pH at 25 °C. Fluorescence spectra were measured from 550 to 750 nm with a 2 nm step, 800 V detector sensitivity using 400 nm excitation. Fluorescence spectra were recorded using a JASCO J-815 CD/fluorescence spectropolarimeter (JASCO International Co., Ltd., Tokyo, Japan).

### 2.8. CD Measurements

CD spectra of oligonucleotides were recorded using a JASCO J-815 spectropolarimeter (JASCO International Co., Ltd., Tokyo, Japan) equipped with a temperature controller. For each sample, 3 spectral scans were accumulated at 25 °C over wavelengths in the range from 220 to 320 nm. Samples of each oligonucleotide were prepared at a concentration of 8.0 μM in 50 mM KCl, 10 mM potassium phosphate buffer, or 150 mM NaCl, 10 mM sodium phosphate buffer at 6.8 pH using a 0.5 cm path length quartz cuvette with a volume of 1750 µL. Next, samples were prepared by heating the oligonucleotides at 90 °C for 5 min and gradually cooling to room temperature. CD spectra were expressed in the units of molar ellipticity Δɛ (cm^2^ mmol^−1^), without normalization by the number of residues in the oligonucleotide. Spectra were processed and prepared using the Origin 8 Software. 

## 3. Results

### 3.1. Formation of Alternative Structures by Hybridization of G-Rich Oligonucleotide to Target Sequences That Differ by a Single Nucleotide Residue

The proposed method to discriminate between two target RNA sequences that differ by one nucleotide only is schematically presented in Figure 1. Generally, it relies on the formation of alternative structures, i.e., a quadruplex–duplex hybrid (QDH) or duplex with long dangling ends (Dss) (Figure 1D,E), after the binding of G-rich oligonucleotides (Figure 1A) to target sequences that differ by a single U/G change (Figure 1B,C). We designed two model target sequences (Table 1), G^T^ and U^T^, containing 5′–GGGCGGG–3′ or 5′–GGGCUGG–3′ fragments, respectively. The CCC oligonucleotide (Figure 1A, Table 1) is composed of two segments, duplex forming (D) and G-quadruplex forming (Q). The duplex forming segment consists of 14 nt and is complementary to 14 nt fragments of both targets (G^T^ and U^T^, Figure 1B,C) being responsible for the formation of a duplex domain (Figure 1D,E). The G-rich segment of the CCC strand was designed to assemble into a G-quadruplex motif only with the G^T^ target (CCC/G^T^, Figure 1D) but not with U^T^ (CCC/U^T^, Figure 1E).

### 3.2. Evaluation of Secondary Structures of CCC/G^T^ and CCC/U^T^ RNA:RNA Complexes

**Figure 2:**
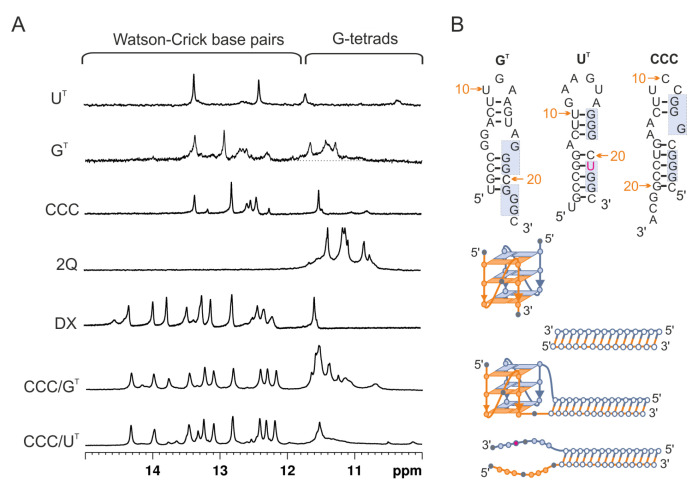
The imino region of the ^1^H NMR spectra of U^T^, G^T^, CCC, 2Q, DX, CCC/G^T^, and CCC/U^T^, recorded in 90% H_2_O/10% D_2_O (*v*/*v*) in the presence of 50 mM KCl, 10 mM potassium phosphate, and 0.1 mM EDTA, pH 6.8; 25 °C (**A**). Proposed secondary structures (2Q, QDH, Dss) and predicted by RNAstructure (hairpins, DX) (**B**).

Both target sequences, G^T^ and U^T^, were hybridized to CCC RNA G-rich oligonucleotides (Table 1) and characterized by several experimental methods. To determine the type of structure formed in the solution, we used ^1^H NMR spectroscopy. In general, imino proton signals associated with Watson–Crick base pairs typically appear at 12–15 ppm, and those between 10.5 and 12 ppm are characteristic of guanosine imino protons involved in G-tetrad formation [70]. The imino region of ^1^H NMR spectra of CCC/G^T^ and CCC/U^T^ are shown in Figure 2A and compared with ^1^H NMR spectra of individual strands of the CCC/G^T^ and CCC/U^T^ molecules (U^T^, G^T^, and CCC), the CGGGCGGGC G-quadruplex (2Q), and the 14 bp duplex (DX). In the presence of K^+^ ions, the NMR spectrum of CCC/G^T^ displayed imino proton signals in both duplex and G-quadruplex regions, and the peak distribution patterns resembled that of the individual components, duplex, and G-quadruplex. This was consistent with the formation of the quadruplex–duplex hybrid structure (QDH) by CCC/G^T^. When CCC RNA oligonucleotide was hybridized to the U^T^ target, only signals characteristic of the imino protons involved in the formation of Watson–Crick base pairs were observed. This suggests that the replacement of one of the guanosine residues by uridine, such as the U^T^ target, can prevent G-quadruplex formation (Figure 1C,E and Figure 2A). For the CCC/U^T^ complex, only signals corresponding to the duplex domain were observed while G-rich fragments remained unstructured (Figure 1E and Figure 2A), indicating the formation of a duplex structure with long dangling ends (Dss).

**Figure 3:**
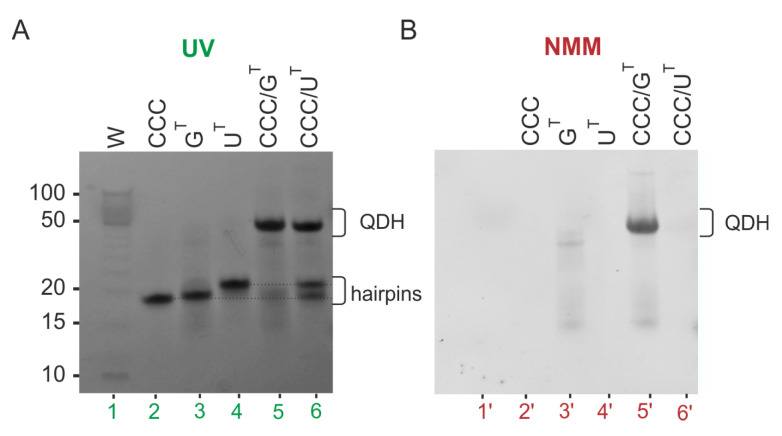
Analysis of the migration of CCC, G^T^, U^T^, CCC/G^T^, and CCC/U^T^ by non-denaturing PAGE 20%; gel was visualized by UV light (254 nm) to detect all RNAs (**A**) and post-stained with NMM solution to detect G4 motifs (**B**).

Next, we performed native polyacrylamide gel electrophoresis (PAGE) in the presence of 50 mM KCl to assess the molecularity of CCC/G^T^ and CCC/U^T^ and to verify structural differences between these two complexes (Figure 3). The bands were first visualized by UV shadowing at 254 nm and immediately after electrophoresis were stained with NMM, a dye that exhibits significantly increased fluorescence only upon binding to parallel G-quadruplexes [71,72]. The first lane in Figure 3 corresponds to DNA markers (10–100 bp), and lanes 2–6 correspond to CCC, G^T^, U^T^, CCC/G^T^, and CCC/U^T^, respectively. The mobilities of the bands corresponding to oligonucleotides CCC, G^T^, and U^T^ (lanes 2–4) were in agreement with the formation of the hairpin structures as predicted by the RNAstructure software (Figure 2B). As expected, the main bands in lanes 5 and 6 migrated similarly to that of the 50 bp DNA marker, indicating the formation of bimolecular structures. In lane 6, bands of non-associated strands of CCC and U^T^ were observed. After staining with NMM, only the band corresponding to CCC/G^T^ (Figure 3, lane 5′) was clearly visible, thus supporting the formation of the G-quadruplex domain.

The binding of NMM to the G-quadruplex domain of CCC/G^T^ suggested its parallel topology [71,72], which was further confirmed by the presence of a characteristic positive band at ~265 nm and a negative band at 240 nm in the CD spectrum (Figure S2). The CD spectrum of CCC/U^T^ differed from that of CCC/G^T^ only in the intensity of the band at ~265 nm. Unfortunately, CD spectra of parallel G-quadruplexes are very similar to those of A-form duplex structures. As a result, they have little use in structural analysis of RNA quadruplex–duplex hybrids [73]. 

The thermal stabilities of CCC/G^T^ and CCC/U^T^ structures were determined by analyzing UV-melting profiles at 260 and 295 nm in the presence of potassium cations (Table 2, Figure S3). The melting temperature of CCC/G^T^ determined at 260, and 295 nm was 69.4 °C and 68.8 °C, respectively (Table 2, Figure S3). The profile of the melting curve at 295 nm did not reflect the typical reverse sigmoid. This resulted from the overlapping of two opposing effects, hypochromic and hyperchromic, for the melting of G-quadruplex and duplex motifs at this wavelength (Figure S3). A similar profile of the melting curve at 295 nm was observed previously for the RNA hairpin structure in equilibrium with the G-quadruplex, but this phenomenon was not discussed [74]. The melting temperature of the CCC/U^T^ duplex determined at 260 nm was 61.0 °C. The higher stability of the CCC/G^T^ hybrid relative to the CCC/U^T^ duplex (ΔT_m_ = 8.4 °C) confirmed the G4-dependent stabilization effect.

### 3.3. Evaluation of Secondary Structures of DNA-CCC/G^T^ and DNA-CCC/U^T^ Complexes

Next, we compared the tendency of G-rich RNA and DNA molecules to form QDH and Dss structures. We hybridized the DNA equivalent of the CCC sequence, DNA-CCC (Table 1), with G^T^ and U^T^ RNA targets in the presence of potassium cations.

**Figure 4:**
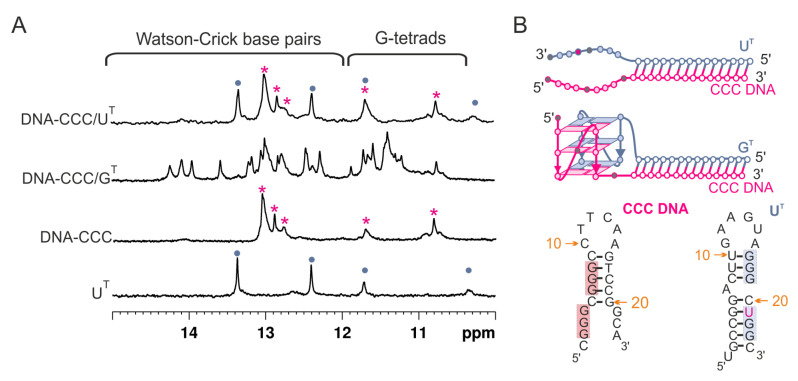
The imino region of the ^1^H NMR spectra of DNA-CCC/U^T^, DNA-CCC/G^T^, DNA-CCC, and U^T^, recorded in 90% H_2_O/10% D_2_O (*v*/*v*) in the presence of 50 mM KCl, 10 mM potassium phosphate, and 0.1 mM EDTA, pH 6.8; 25 °C (**A**). Red stars—imino signals of DNA-CCC, blue dots—imino signals of U^T^. Proposed secondary structures (2Q, QDH, Dss) and predicted by RNAstructure (hairpins, DX) (**B**).

Figure 4A shows the ^1^H NMR spectra of two DNA:RNA hetero-complexes: DNA-CCC/U^T^ and DNA-CCC/G^T^. The spectrum of the DNA-CCC/G^T^ displayed signals both in duplex (11.5–15 ppm) and G-quadruplex (10.5–12 ppm) regions, indicating the formation of a hybrid quadruplex–duplex structure. The ^1^H NMR spectrum of DNA-CCC/U^T^ showed that signals in the region of 10.5–12 ppm characteristic of G-quadruplex formation were missing, thus suggesting the formation of a Dss structure. However, comparison of the ^1^H NMR spectrum of DNA-CCC/U^T^ with DNA-CCC and U^T^ clearly showed that it was a sum of signals characteristic of two separate strands, i.e., DNA-CCC and U^T^, thus suggesting that the structure of a DNA: RNA heteroduplex with dangling ends was not formed (Figure 4A,B).

**Figure 5:**
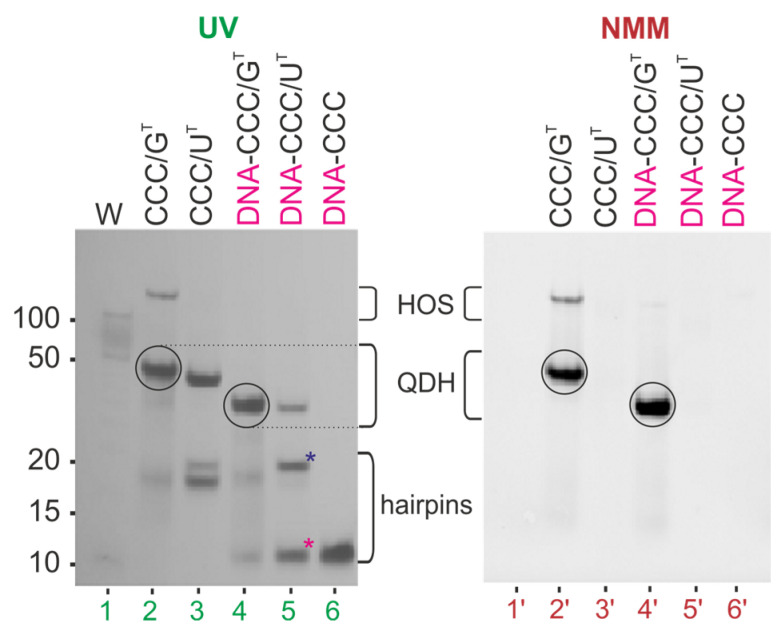
Analysis of the migration of CCC/G^T^, CCC/U^T^, DNA-CCC/G^T^, DNA-CCC/U^T^, and DNA-CCC by non-denaturing PAGE 20%; gel was visualized by UV light (254 nm) to detect all RNAs and post-stained with NMM solution to detect G4 motifs. Red star—DNA-CCC, blue star—U^T^, HOS—higher-order structure, QDH—quadruplex-duplex hybrid. The first lane corresponds to the DNA ladder marker in the range of 10 to 100 bp (W).

Figure 5 compares the electrophoretic mobility of hetero-complexes DNA-CCC/G^T^, DNA-CCC/U^T^ (lanes 4–5), and single-strand DNA-CCC (lane 6) relative to RNA homo-complexes CCC/G^T^ and CCC/U^T^ (lanes 2–3). Bands corresponding to bimolecular structures DNA-CCC/G^T^ and DNA-CCC/U^T^ migrated faster than their RNA:RNA counterparts. The smaller intensity of the band corresponding to DNA-CCC/U^T^ (lane 5) than that of CCC/U^T^ (lane 3), and the presence of unhybridized single-strands DNA-CCC and U^T^ in lane 5, were indicative of a poor hybridization of DNA to RNA strands. When the gel was post-stained with NMM, fluorescence bands were observed only for CCC/G^T^ and DNA-CCC/G^T^ and indicated the formation of the G-quadruplex domain in both cases (Figure 5, bands 2ʹ, 4ʹ). Additionally, the presence of a minor band characterized by slow electrophoretic mobility for CCC/G^T^ indicated the formation of higher-order structures (HOS) (Figure 5, lane 2 and lane 2′). An increase in intensity with time was observed for those retarded bands for CCC/G^T^ (Figure S4). This structure could be a dimer of two G-quadruplex domains of QDH structures. The parallel topology of the G4 domain of the DNA-CCC/G^T^ hybrid was additionally confirmed by a characteristic CD spectrum (Figure S5). The DNA-CCC/G^T^ structure was less stable than that of CCC/G^T^, as indicated by the comparison of melting temperatures, which were 61.4 and 69.4 °C, respectively (Figure S6, Table 2). Data obtained from the NMR spectra showed that the DNA-CCC/U^T^ complex was not formed at room temperature (Figure 4). Therefore, for further studies, we decided to use RNA G-rich oligonucleotides because they are definitely more stable when hybridized to RNA targets, thus enabling the determination of secondary structures. In addition, the sequential requirements for the formation of RNA quadruplex–duplex hybrids are less known.

### 3.4. Impact of the Chemical Modifications on the Ability to Form Alternate QDH and Dss Structures

**Figure 6:**
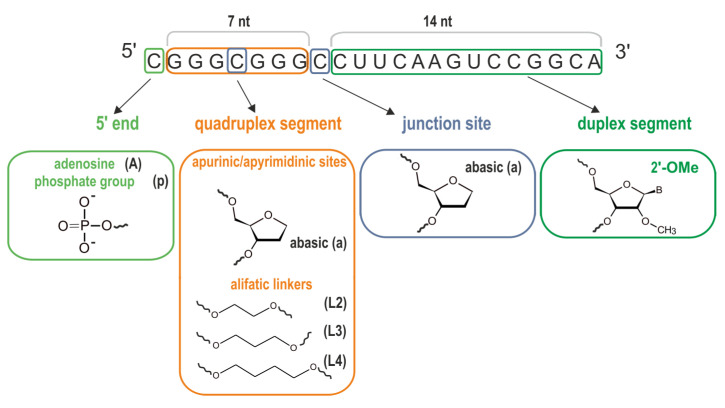
The chemical formulas and the sites of chemical modifications in the QD strand.

The modified G-rich oligoribonucleotides used in this study, and their corresponding names and notations used to describe the bimolecular complexes, are summarized in Table 1. Bolded cytidine residues in the CCC sequence point to the modification sites (Table 1, Figure 6). Non-nucleotide modifications, such as abasic (a), aliphatic 1,2-ethanediol (L2), 1,3-propanediol (L3), and 1,4-butanediol (L4) linkers, were chosen to study their effect on the formation of the G-quadruplex domain. It was previously reported that the presence of abasic residues and long aliphatic linkers in the loop region promoted and stabilized the formation of parallel G-quadruplex topologies [16,17,75]. In turn, the presence of a phosphate group (p) or additional nucleotide residues at the 5′-end should prevent dimerization of G-quadruplexes [76,77]. A 2′O-Me modification in a duplex segment was used to estimate its potential to stabilize QDH and Dss structures. 

**Figure 7:**
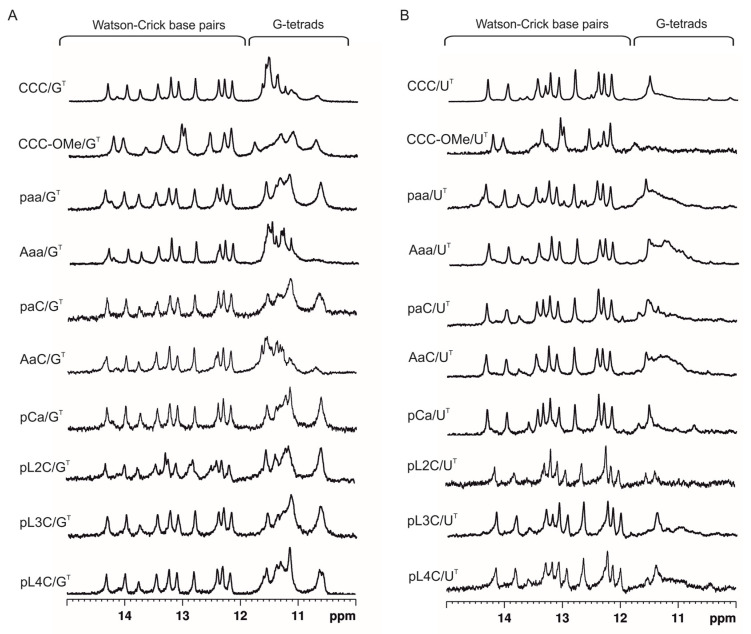
Comparison of imino region of the ^1^H NMR spectra of CCC/G^T^, CCC-OMe/G^T^, paa/G^T^, Aaa/G^T^, paC/G^T^, AaC/G^T^, pCa/G^T^, pL2C/G^T^, pL3C/G^T^, pL4C/G^T^ (**A**) and CCC/U^T^, CCC-OMe/U^T^, paa/U^T^, Aaa/U^T^, paC/U^T^, AaC/U^T^, pCa/U^T^, pL2C/U^T^, pL3C/U^T^, pL4C/U complexes (**B**) at 25 °C in the presence of 50 mM KCl, 10 mM potassium phosphate, 0.1 mM EDTA, pH 6.8.

To determine the impact of the chemical modifications on the formation and stability of G-quadruplex motifs, we compared the ^1^H NMR spectra of unmodified and modified G-rich oligoribonucleotides hybridized to G^T^ and U^T^ targets in the presence of K^+^ and Na^+^ cations. The corresponding ^1^H NMR spectra of QD/G^T^ complexes recorded in buffers containing K^+^ or Na^+^ cations are presented in Figure 7 and Figure S6, respectively. All modified G-rich oligoribonucleotides after hybridization to G^T^ target display the imino peak patterns typical of the structure containing Watson–Crick and Hoogsteen-type hydrogen bonds. In general, all these spectra were similar to that of the model CCC/G^T^ hybrid, regardless of the type of modification, their modification site, and the type of ions present in the solution, K^+^ or Na^+^. For two complexes in a solution containing potassium ions, AaC/G^T^ and Aaa/G^T^, a slightly different pattern was observed in the Hoogsteen region, which could be due to the presence of adenosine residues at the 5′-end in AaC and Aaa strands (Figure 7). 

When modified G-rich oligoribonucleotides were hybridized to the U^T^ target (QD/U^T^ complexes) in the presence of K^+^, in addition to the imino protons from Watson–Crick base pairs, broad resonances were observed in the 10.5–12.0 ppm region for several complexes containing modified residues (for instance: paa/U^T^, Aaa/U^T^, pL4C/U^T^, AaC/U^T^, or pCa/U^T^) (Figure 7). In sodium phosphate buffer, a broad resonance suggesting the presence of a G4 motif was clearly visible only for molecules containing two abasic residues (paa/U^T^, Aaa/U^T^), whereas for AaC/U^T^ these G4 imino signals were very broad (Figure S7). These results suggest that the incorporation of chemical modifications into G-rich strands can promote the formation of structures other than Dss after hybridization to the U^T^ strand. These structures may contain G-quadruplex motifs instead of dangling ends.

**Figure 8:**
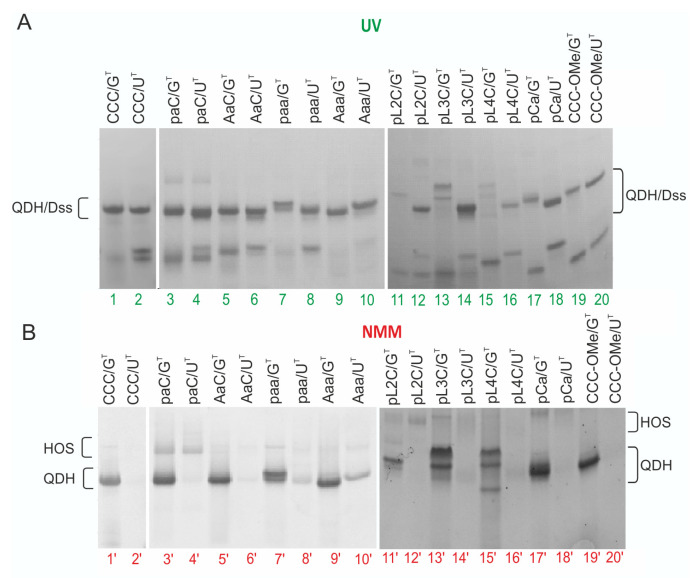
Analysis of the migration of CCC/G^T^, CCC/U^T^, and chemically modified QD/G^T^ and QD/U^T^ complexes in potassium phosphate buffer by non-denaturing PAGE 20%; gels were visualized by UV light (254 nm) to detect all RNAs (**A**) and post-stained with NMM solution to detect G4 motifs (**B**).

The effect of the chemical modification on the formation of QDH and Dss structures was further analyzed by native PAGE (Figure 8, UV and NMM visualization). In the presence of K^+^ cations, the migration rate of bands corresponding to modified QD/G^T^ complexes (Figure 8A, lines 3–20) was in agreement with the formation of the CCC/G^T^ quadruplex–duplex hybrid (Figure 8A, lines 1) and these bands were efficiently stained with NMM (Figure 8B, lines 1′). Slower migration of pL2C/G^T^, pL3C/G^T^, and pL4C/G^T^ bands probably reflected the reduced negative charge of these hybrids resulting from the introduction of aliphatic linkers. Moreover, for QD/G^T^ complexes comprising two abasic residues or L3 and L4 aliphatic linkers and a phosphate group at the 5′-end (paa/G^T^, pL3C/G^T^, pL4C/G^T^, Figure 8B, lines: 7′, 13′, 15′ respectively), the main bands were split into two bands with similar fluorescence intensity after staining with NMM. The presence of two bands suggested the formation of two different conformers. For this parallel G-quadruplex motif, there are two forms possible that differ in the directionality of the hydrogen bonds within the G-tetrads (Figure S8). Additionally, similarly to CCC/G^T^ molecule, the presence of minor bands characterized by slow electrophoretic mobility for most modified QD/G^T^ complexes indicated the formation of higher-order structures (HOS) (Figure 8B).

A comparison of the migration of QD/G^T^ hybrids containing a phosphate group or adenosine at the 5′-end of QD (Figure 8B, band 3′ vs. 5′ and 7′ vs. 9′) showed that the presence of adenosine prevented the dimerization of two G-quadruplex units of the QDH structure more than the phosphate group. The QD/U^T^ complexes containing non-nucleotide linkers migrated with a mobility similar to that of the model CCC/U^T^ (Figure 8). Most of the bands corresponding to bimolecular structures did not stain with NMM (even lines), confirming the formation of duplexes with dangling ends as the dominant form. However, for paa/U^T^ (lane 8′), Aaa/U^T^ (lane 10′) low-intensity fluorescence bands were observed after staining with NMM (Figure 8B). To confirm the formation of a G-quadruplex motif in paa/U^T^ and Aaa/U^T^, we performed an experiment in which we compared fluorescence emissions after adding equimolar ratios of representative QD/G^T^ and QD/U^T^ to a solution of NMM (Figure S9). The intensity of the fluorescence signal of NMM and NMM after the addition of CCC/U^T^ (1:1) was similarly low, indicating a weak affinity of NMM towards CCC/U^T^. For NMM complexes with Aaa/G^T^ and paa/G^T^, we observed a moderate 7- to 9-fold enhancement of NMM fluorescence emission, whereas, for Aaa/U^T^ and paa/U^T^, a 4–6.5-fold enhancement of NMM fluorescence emission was determined. The intensities of the fluorescence signals of the NMM in complex with Aaa/U^T^ and paa/U^T^ were significantly higher compared to those obtained for NMM in complex with CCC/U^T^ duplex. These results confirmed the selective binding of NMM to the non-canonical G-quadruplex domain of Aaa/U^T^ and paa/U^T^.

Additionally, we recorded ^1^H NMR spectra after the addition of one equivalent of NMM to NMR samples containing paa/G^T^, paa/U^T^, or Aaa/U^T^ (Figure S10). Ligands binding to paa/G^T^ resulted in a significant broadening of the imino signals corresponding to G-tetrads and the appearance of signals from the NMM ligand in the 10.3–9.0 ppm region (Figure S10a). NMM-dependent broadening of the imino signals was also observed in the ^1^H NMR spectra of Aaa/U^T^ and paa/U^T^ (Figure S10b,c). These results strongly supported data obtained from native PAGE and fluorescence experiments, indicating that a noncanonical G-quadruplex domain could form in the Aaa/U^T^ and paa/U^T^ molecules. The formation of G-quadruplex motifs in Aaa/U^T^ and paa/U^T^ structures, which was observed in the presence of K^+^ ions, was also observed in native PAGE performed in the presence of Na^+^ ions (Figure S11, lines 5′ vs. 6′ and 7′ vs. 8′).

To further characterize QD/G^T^ and QD/U^T^ complexes, we analyzed their UV-melting profiles in solutions containing K^+^ or Na^+^ ions. The apparent melting temperatures determined based on measurements at 260 nm are collected in Table 2 and Table S1 and presented graphically in Figures S12a,b and S13a,b (melting curves at 260 and 295 nm). For several quadruplex–duplex hybrid structures, accurate determination of T_m_ at 295 nm was not possible (Figures S12 and S13). However, analysis of melting profiles at 295 nm was used to estimate the stability of the G-quadruplex motif and to confirm the formation of QDH or Dss structures in both K^+^ and Na^+^ solutions. Profiles of melting curves at 295 nm indicated the presence of G-quadruplex motifs for all QD/G^T^ hybrid molecules and for paa/U^T^, Aaa/U^T^, and AaC/U^T^ in potassium phosphate buffer (Figures S12a and S13a). All QD/G^T^ structures had comparable T_m_ values in the range of 67.4–69.0 °C, regardless of the type and site of modification, and were very close to that of CCC/G^T^ (69.4 °C) (Table 2). The only exception was observed for 2’OMe modification, which is well known to stabilize RNA duplex structures (Table 2). In addition, we confirmed that the introduction of non-nucleotide modifications does not affect the kinetics of QDH structure formation (no hysteresis was observed, K^+^, data not shown). In general, in potassium phosphate buffer, all QD/G^T^ structures were more stable than the corresponding QD/U^T^ complexes; their T_m_ values were higher at least by 6.0 °C. For paa/U^T^, Aaa/U^T^, and AaC/U^T^, molecules for which the presence of noncanonical G-quadruplex motifs was suggested, analysis of the melting curves at 295 nm indicated that these motifs were less stable than their QD/G^T^ counterparts (in average by 7.25 °C). Apparent T_m_ values determined for the quadruplex–duplex hybrid structures in the presence of sodium cations (Table S1) were comparable to the corresponding melting temperatures of duplexes with dangling ends. This was due to the increased stability of the duplex motif ascribed to the different ionic strengths of the buffers (60 mM K^+^ versus 160 mM Na^+^). By comparison, the exchange of K^+^ with Na^+^ caused a significant decrease in the stability of the G-quadruplex domain of QD/G^T^, which could be estimated from the melting curve recorded at 295 nm and in ^1^H NMR spectra (Tables S1, S12b, and S14b).

Different thermal stabilities of duplex and G-quadruplex motifs depending on the solution conditions were best illustrated by ^1^H NMR spectra recorded as a function of temperature. Figure S14 shows the ^1^H NMR spectra of CCC/G^T^, paa/G^T^, and pL3C/G^T^ obtained in the range of 25–75 °C in buffers containing 60 mM K^+^ and 160 mM Na^+^ (Figure S14a,b). In the potassium environment, with increasing temperature, Watson–Crick imino signals disappeared faster than those from G-tetrads. Moreover, the stability of the G-quadruplex motifs appeared to be higher for molecules containing modified residues. By comparison, in ^1^H NMR spectra recorded in the presence of sodium cations, the G4 signals tend to disappear at a faster rate compared to Watson–Crick base pairs.

Although CD spectra of RNA G-quadruplexes were very similar to those of A-form duplexes, the presence of a clear positive peak at 260 nm and a negative peak at 240 nm for all QD/G^T^ complexes studied (Figure S15, Na^+^, K^+^) verified the parallel topology of their G-quadruplex motifs. For QDH structures containing chemically modified G-rich strands, an increase in the intensities of the Cotton effects at 265 nm was observed compared to CCC/G^T^. As expected, the shape of the CD spectrum of the CCC/U^T^ duplex with dangling ends was similar to that of the quadruplex–duplex hybrids; however, the bands at 265 nm were of lower intensity than the spectra of the corresponding QDH structures (Figures S2 and S15). For paa/U^T^ and Aaa/U^T^ molecules, the observation of increased intensity of the ellipticity values in CD spectra was consistent with the presence of the G-quadruplex motif in their structures (Figure S16).

### 3.5. Influence of CCC Oligonucleotide with Covalently Attached G4 Ligand on the Formation and Stability of QDH and Dss Structures

Several small-molecule ligands are known to specifically bind and stabilize G-quadruplexes and are considered to be promising therapeutic targets. However, few ligands are known that are specific to a given G-quadruplex topology [78]. Therefore, we decided to covalently attach a well-known o-BMVC ligand to the CCC oligoribonucleotide and investigate its impact on the secondary structure formation after hybridization to the target sequence G^T^ or U^T^. We chose the o-BMVC ligand due to its properties, which are ideal for the purpose of our study, i.e., a large difference in fluorescent intensity after binding to G4 structures or duplexes [79,80]. Derivative of o-BMVC, propionic acid NHS ester (o-BMVC-C3-NHS) was made in-house [52,81] and then attached to the 5′-end of the CCC molecule via a C6-aminolinker (o-BMVC-C3-CCC) (Figure S17).

**Figure 9:**
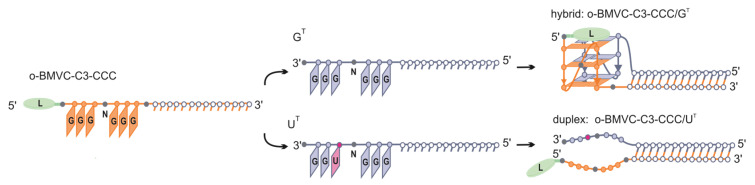
The recognition of G^T^ and U^T^ targets by a model L–CCC oligonucleotide resulting in the formation of a ligand–quadruplex–duplex structural hybrid (L–CCC/G^T^) or duplex with long dangling ends (L–CCC/U^T^).

**Figure 10:**
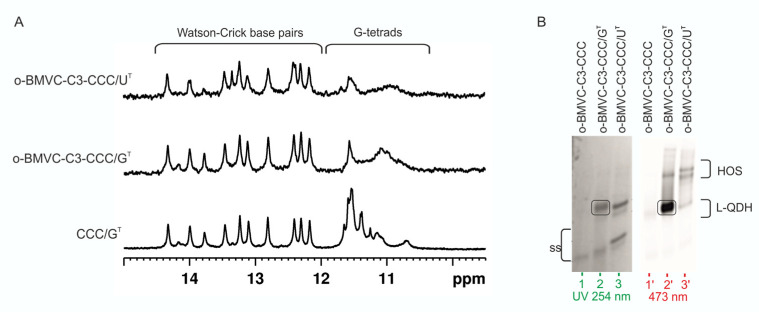
The imino region of the ^1^H NMR spectra of o-BMVC-C3-CCC/G^T^, o-BMVC-C3-CCC/U^T^, and CCC/G^T^ recorded in 90% H_2_O/10% D_2_O (*v*/*v*); 25 °C (**A**). Migration of o-BMVC-C3-CCC, o-BMVC-C3-CCC/G^T^, and o-BMVC-C3-CCC/G^T^ in non-denaturing PAGE 20%. Gel was visualized by UV light (254 nm) to detect all RNAs (**B**, **left**), and the same gel was exposed to 473 nm light to visualize the RNA structure adopting the ligand–quadruplex domain (**B**, **right**) in the presence of 50 mM KCl, 10 mM potassium phosphate and 0.1mM EDTA, pH 6.8.

The structure and stability of complexes of o-BMVC-C3-CCC (L-CCC, Table 1) annealed to G^T^ or U^T^ (Figure 9) were investigated using ^1^H NMR, UV, CD, and fluorescence spectroscopy. ^1^H NMR spectra of CCC/G^T^, o-BMVC-C3-CCC/G^T^, and o-BMVC-C3-CCC/U^T^ are shown in Figure 10A. The presence of imino signals in the o-BMVC-C3-CCC/G^T^ spectrum in the region typical of Watson–Crick base pairs and of G-tetrads confirmed the formation of both duplex and G-quadruplex domains (Figure 10A). Furthermore, a significant broadening of signals only in the 10.5–12 ppm region indicated that ligand bounds selectively to the G-quadruplex motif. In the ^1^H NMR spectrum of the o-BMVC-C3-CCC/U^T^, signals corresponding to the duplex domain remained sharp, but a broad signal of low intensity additionally appeared in the 10.5–12 ppm region. This suggested that o-BMVC-C3 could possibly interact nonspecifically with G-rich dangling ends.

Structures of o-BMVC-C3-CCC/G^T^ and o-BMVC-C3-CCC/U^T^ were further investigated using native PAGE gel electrophoresis. The migration of o-BMVC-C3-CCC/G^T^ (Figure 10B, lane 2) and o-BMVC-C3-CCC/U^T^ (Figure 10B, lane 3) visualized in UV light appeared to be undoubtedly slower than that of the o-BMVC-C3-CCC single-strand (Figure 10B, lane 1), clearly showing the presence of bimolecular complexes. After exposure of the gel to 473 nm light, strong fluorescence was observed only for o-BMVC-C3-CCC/G^T^ when visualized at 532 nm. The large difference in fluorescence intensity observed between o-BMVC-C3-CCC/G^T^ and o-BMVC-C3-CCC/U^T^ molecules confirmed the formation of a G-quadruplex domain only in the o-BMVC-C3-CCC/G^T^ hybrid (Figure 10B). The presence of a weak fluorescent band corresponding to the o-BMVC-C3-CCC/U^T^ conjugate supported the earlier observation from analysis of the ^1^H NMR spectra, suggesting that the covalently attached ligand o-BMVC-C3 could interact nonspecifically with G-rich dangling ends. Additionally, the formation of higher-order species was observed for both complexes. To determine the impact of the covalently attached ligand on the thermal stabilities of the studied molecules, we performed UV-melting experiments in the presence of potassium cations. The melting temperature obtained for o-BMVC-C3-CCC/G^T^ (T_m_ = 72.1 °C) was 2.7 °C higher than that for CCC/G^T^ (Figure S18). In turn, attachment of the G4 ligand to CCC/U^T^ did not affect its thermal stability; T_m_ values of o-BMVC-C3-CCC/U^T^ and CCC/U^T^ were the same within experimental error, 61.2 °C and 61.0 °C, respectively. 

The CD spectra of o-BMVC-C3-CCC/G^T^ and o-BMVC-C3-CCC/U^T^ are compared to the CD spectrum of CCC/G^T^ in Figure S19. The presence of a strong maximum at 265 nm indicated that the G-quadruplex domain of o-BMVC-C3-CCC/G^T^ maintained a parallel topology. As can be seen, the intensity of this band was significantly higher than that of o-BMVC-C3-CCC/U^T^, which further supported the formation of a stable G-quadruplex domain. By comparison, the lower intensity of this band observed for CCC/G^T^ relative to o-BMVC-C3-CCC/G^T^ suggested that in a molecule without an attached ligand, the G-quadruplex domain could be less structured. 

## 4. Discussion

### 4.1. Quadruplex–Duplex Hybrid Structures (RNA vs. DNA)

Thousands of RNA and DNA sequences that are able to form stem-loop-containing G-quadruplexes have been identified in the human genome and transcriptome using bioinformatic methods [39]. Systematic studies of the unimolecular DNA QDH structures comprising a hairpin or multiple stems in place of at least one of the loops suggest that the junction between the G-quadruplex and duplex motifs is characteristic of a particular G4 topology: parallel, antiparallel, or hybrid [41,44]. Recently, QDH structures with a duplex stem incorporated into a bulge of a G-quadruplex or structures with a duplex stem build from the G4 flanking regions have also been demonstrated [45,46,47,82]. QDH structures identified in RNA aptamers (Spinach, Mango, and FMRP-binding) were found to have unique G-core architectures [82]. By comparison, all known bimolecular RNA:RNA or DNA:RNA quadruplex–duplex structures tend to be structurally conservative and are constructed of a parallel G-quadruplex domain and a duplex stem located in the place of an external loop [50,51,52]. This is in agreement with our results showing that all RNA-RNA homohybrids studied in this work and DNA-CCC/G^T^ heterohybrids adopt this architecture (Figure 1D, Figure 2B and Figure 4B). This structural conservatism of bimolecular RNA:RNA or DNA:RNA QDHs allows stable hybrid structures to be predicted, and this could help in the future to design drugs that selectively stabilize G-quadruplex motifs.

### 4.2. Stability of QDH and Dss Structures

Although a type of G-rich oligonucleotide (DNA or RNA) did not change the structural preferences of QD/G^T^ complexes, the use of DNA strands instead of RNA reduced the stability of DNA-CCC/G^T^ heterohybrid by 8 °C. This destabilizing effect was even more pronounced in the case of the DNA-CCC/U^T^ complex. We showed that this complex was unstable and did not form at room temperature. In the presence of potassium cations, the thermal stability of the examined quadruplex–duplex hybrid structures increased by ~8 °C compared to the corresponding duplexes with dangling ends. Due to the significant destabilization of the G-quadruplex domain (Figure S14b) in a solution containing Na^+^ ions, this duplex domain was responsible for the stability of the QDH. The other important factor that could influence the stability and/or structure of G-quadruplexes was the incorporation of non-nucleoside modifications, such as an aliphatic linker or abasic residues, into a G-rich region. It was previously shown that the replacement of TTA loops with aliphatic linkers of different lengths in the human telomeric repeat sequences (GGGTTA)_3_G_3_ resulted in a conversion of G-quadruplex topology from antiparallel to parallel [16]. The stability of the modified G-quadruplexes was strongly correlated with the length of the linker in the order 1,8-octanediol > hexaethylene glycol > 1,3-propanediol. Interestingly, the G-quadruplex bearing the 1,8-octanediol loop was found to adopt several stable conformations, including the formation of the two-layer G-quadruplex structure as proposed by the authors. Replacement of one nucleoside loop with abasic residue resulted in the most stable DNA G-quadruplexes. Furthermore, it was shown that RNA G-quadruplexes containing 1 nt external loops are the most stable [83]. In the present study, the replacement of a single nucleoside loop with a non-nucleotide modification led to the formation of stable QDH structures irrespective of the modification type or the length of the linker. When the external loop was replaced by L3 or L4 aliphatic linkers, the formation of two bands corresponding to two conformers of pL3C/G^T^ and pL4C/G^T^ quadruplex–duplex hybrids were observed in the native PAGE experiment. The formation of two conformers was also detected for the paa/G^T^ complex containing abasic residues located in loop and junction positions. It has been previously reported that base composition proximal to the junction between duplex and G-quadruplex motifs can also play an important role in both the structure and stability of the hybrids. For example, when the terminal C:G base pair was substituted with a less stable T:A base pair in a junction, two bands appeared in gel electrophoresis, probably due to two different conformations present in solution [84]. In general, non-nucleotide linkers can contribute to the increased flexibility of the loop or influence the interaction between the loop and G-tetrads. Our results indicate that the replacement of the loop or junction residues with a flexible linker did not change the structure and stability of QDHs. Furthermore, flexible linkers appeared to have a high propensity to form external loops. When the G4-selective ligand was conjugated to the G-rich oligonucleotide, a moderate increase in stability was observed for o-BMVC-C3-CCC/G^T^ relative to CCC/G^T^. In contrast, the presence of the non-nucleotide modifications or G4 ligand did not affect the stability of duplexes with dangling ends.

### 4.3. Formation of Non-Canonical Hybrid Structures

Here, we demonstrated that one nucleotide change in a G-rich oligonucleotide (change from 5′–GGGCGGG–3′ to 5′–GGGCUGG–3′) prevented the formation of the G-quadruplex domain in the CCC/U^T^ complex. For the CCC/U^T^ molecule, we considered the possibility of the formation of structures other than Dss. For this purpose, we used the RNAstructure software, which suggested several stable secondary structures with additional canonical Watson-Crick or non-canonical base pairs (Figure S20). However, analysis of the imino region of the ^1^H NMR spectrum (Figure 2) indicated that the duplex with the dangling ends was a dominant form of CCC/U^T^. The number of imino signals observed in the ^1^H NMR spectrum precluded the formation of additional stable base pairs in the structure of CCC/U^T^. However, in the ^1^H NMR spectra of paa/U^T^ and Aaa/U^T^, we surprisingly noticed an appearance of signals indicating the formation of G-tetrads, even in the solution containing Na^+^ cations. The formation of this G-quadruplex motif was also confirmed by the observation of low-intensity fluorescence bands in native PAGE after staining with NMM. UV-melting experiments showed that thermal stabilities of paa/U^T^ and Aaa/U^T^ were lower than those determined for their counterparts paa/G^T^ and Aaa/G^T^. These results suggest that G-quadruplex domains in paa/U^T^ and Aaa/U^T^ could be composed of two G-tetrads, or two G-tetrads and one mixed GGGU tetrad (Figure S21). The binding of NMM to this atypical G-quadruplex motif was additionally confirmed by ^1^H NMR experiments and an increased emission in fluorescence spectra after the addition of paa/U^T^ or Aaa/U^T^ to a solution containing NMM. It was previously reported that the hairpin in dilute solution could undergo the transition to a G-quadruplex containing one mixed GGUU tetrad. This type of G-quadruplex exhibited reduced thermal stability and fluorescence intensity after NMM binding [29]. In summary, our data suggest that the formation of G-quadruplexes is possible even when the sequences lack regular G-tracts, which were not predicted by algorithms developed to search for putative G-quadruplex forming sequences. Moreover, the obtained results advance our knowledge regarding the influence of non-nucleotide modifications on the formation of non-canonical G-quadruplexes. In this respect, a recent report should be mentioned that demonstrated that the presence of non-guanosine residues or bulges in the G-core generally destabilized the G-quadruplex structure but nevertheless did not prevent G-quadruplex formation [33,85,86]. Another study has shown that G-quadruplexes with mixed central tetrads were able to compensate for the lack of a G-tetrad in the context of both GTP-binding and peroxidase activity [28]. These data shed light on the possible biological functionality of non-canonical G-quadruplexes. 

### 4.4. Application Potential of G-Rich Oligonucleotides

We have recently shown that the formation of a stable G4 motif on an mRNA template was an effective steric hindrance for ribosomes and results in stalling and inhibition of protein synthesis. The silencing activity of oligoribonucleotides modified with 1,6-hexanediol and an abasic linker toward the EGFR mRNA target appeared to be at least 20% higher than for classical 16 nt antisense oligonucleotides [52]. Several studies indicated that a correlation exists between G-quadruplex stability and efficacy of gene expression silencing [50,87,88]. Our data revealed that RNA:RNA QDH was more stable, by 8 °C, than the corresponding DNA:RNA heterohybrid. Additionally, we demonstrated that abasic residues and L2-L4 aliphatic linkers could perfectly mimic external loops. A previous study indicated that the incorporation of the methylene unit (L1) into the TBA sequence could enhance the lifetime of the G-quadruplex in blood [89]. Another study has shown that the presence of a long aliphatic linker between G-tracts can prevent the hybridization of a G-rich oligonucleotide to a C-rich sequence [16]. We strongly believe that chemically modified RNA G-rich antisense oligonucleotides forming stable quadruplex–duplex hybrid structures with mRNA have significant application potential [52]. The ability of these G-rich oligonucleotides to recognize a single nucleotide change in the target sequences in a secondary structure-dependent manner (Figure 1) increases their attractiveness. For therapeutic purposes, the advantages of using G-rich oligonucleotides with a duplex forming segment that is complementary to the target sequence rely on the ability to guide these oligonucleotides to a specific mRNA site. Reviewing the group of representative human mRNAs, we found that over 90% of mRNA annotated in the human genome (RefSeq) contain at least one potential target sequence composed of two neighboring blocks of consecutive guanosine residues separated by one or two non-guanosine residues: HGGGH(H)GGGH, HGGGGH(H)GGGGH (H denotes A, C or T). With the use of mRNA sequences retrieved from the RefSeq database (www.ncbi.nlm.nih.gov/refseq) and the IGV tool (www.broadinstitute.org/igv (accessed on 31th October 2019)), we identified potential target sequences in protooncogenes EGFR, KRAS, HER2, and genes implicated in human Mendelian dominant disorders, such as PTPN11 and FGF23. The G-rich oligonucleotides may also be considered for allele-specific targeting. For example, the well-recognized cancer-driving EGFR mutation, L858R (c.2573T>G), introduces a 5′–GGGCUGG–3′ to 5′–GGGCGGG–3′ change in the sequence of the EGFR mRNA, creating a potential target for G-rich oligonucleotides [90]. L858R next to exon 19 in-frame deletions are the most frequent EGFR mutations (cumulatively accounting for ~90% of all EGFR mutations), well recognized as biomarkers of targeted therapy of non-small cell lung cancer and other cancers with the use of EGFR-specific tyrosine kinase inhibitors. 

### 4.5. G4 Ligand Conjugated to Oligonucleotide

Ligands interacting with G4s have attracted significant attention as potential anticancer therapeutics [91,92]. In view of the high number of G-quadruplex forming sequences identified in a human genome, the possibility of non-specific interactions with G4 ligands cannot be neglected [93]. The attractiveness of G4 ligands may be enhanced by determining a means to increase their ability to specifically bind and stabilize G4 structures. In this paper, we presented the concept of covalent attachment of G4 ligands to G-rich oligoribonucleotides. The use of such conjugates creates the possibility of inducing and stabilizing the bimolecular G-quadruplexes on the G-rich mRNA template in a sequence-specific manner. We showed recently that oligonucleotides with a covalently attached fluorescent carbazole derivative (o-BMVC-Q-ASO) recognized the target site on EGFR mRNA comprising two G-tracts separated by 28 residues by forming a ligand–quadruplex–duplex hybrid structure. The observed level of green fluorescence of an o-BMVC moiety in three different cancer cells correlated well with the amount of EGFR mRNA [52]. We believe that the formation of QDH or Dss structures is dependent on a single nucleotide change in the target sequence (Figure 1), and the possibility to selectively stabilize the G-quadruplex domain by attaching the G4 ligand (Figure 9) may become an attractive alternative therapy for patients with an EGFR-L858R mutation. Furthermore, o-BMVC-C3-oligonucleotides can be used as fluorescent hybridization probes to visualize the single nucleotide EGFR-L858R mutation of mRNA. The use of sequence-guided G4 ligands acting as a G4 stabilizer can minimize the problem of the dynamic nature of the RNA G-quadruplex by slowing the action of G4-helicases [60,94,95]. In our opinion, the results obtained in this work provide new perspectives to solve the problem of multi-target binding of G4 ligands.

## 5. Conclusions

In this article, we proposed a method to discriminate between two similar target RNA sequences, G^T^ and U^T^, that differ in one nucleotide only, based on the formation of alternative structures, i.e., quadruplex–duplex hybrids or duplexes with dangling ends, respectively. We designed DNA and RNA G-rich oligonucleotides with the ability to trigger the formation of a G-quadruplex motif only when hybridized to the G^T^ target and leaving an unstructured G-rich fragment when hybridized to the U^T^ sequence. We also showed that the replacement of RNA G-rich oligonucleotides with DNA decreased the thermal stability of the quadruplex–duplex hybrid structure and the duplex with dangling ends. Subsequently, we demonstrated that abasic residues and aliphatic linkers can mimic the external loops of bimolecular RNA G-quadruplexes. The quadruplex–duplex hybrid structures containing these non-nucleotide modifications exhibited similar stability as their unmodified counterparts. Unexpectedly, we noticed that the presence of two abasic modifications in G-rich strands induced the formation of non-canonical G-quadruplexes after hybridization to the U^T^ target. Finally, RNA G-rich oligonucleotides with a covalently attached carbazole derivative, o-BMVC-C3, were shown to selectively bind and stabilize a G-quadruplex domain of QDH. The obtained results advance our knowledge and ability to predict structures adopted by G-rich sequences and can be used as a starting point to design anti-EGFR G-rich antisense oligonucleotides. Moreover, o-BMVC-C3 covalently attached to an oligonucleotide allows fluorescent probe visualization of sequences containing two GGG tracts. 

## Figures and Tables

**Table 1:** List of RNA and DNA Sequences (5′→3′) Analyzed in This Work.

Name	RNA G-Rich Oligonucleotide Sequences (QD) 5′-3′	Bimolecular Complexes
CCC	**C**-GGG-**C**-GGG-**C**-CUUCAAGUCCGGCA	CCC/G^T^, CCC/U^T^
CCC-OMe	C-GGG-C-GGG-C-(CUUCAAGUCCGGCA)2′OMe	CCC-OMe/G^T^, CCC-OMe/U^T^
paa	p-GGG-a-GGG-a-CUUCAAGUCCGGCA	paa/G^T^, paa/U^T^
Aaa	A-GGG-a-GGG-a-CUUCAAGUCCGGCA	Aaa/G^T^, Aaa/U^T^
paC	p-GGG-a-GGG-C-CUUCAAGUCCGGCA	paC/G^T^, paC/U^T^
AaC	A-GGG-a-GGG-C-CUUCAAGUCCGGCA	AaC/G^T^, AaC/U^T^
pCa	p-GGG-C-GGG-a-CUUCAAGUCCGGCA	pCa/G^T^, pCa/U^T^
pL2C	p-GGG-L2-GGG-a-CUUCAAGUCCGGCA	pL2C/G^T^, pL2C/U^T^
pL3C	p-GGG-L3-GGG-a-CUUCAAGUCCGGCA	pL3C/G^T^, pL3C/U^T^
pL4C	p-GGG-L4-GGG-a-CUUCAAGUCCGGCA	pL4C/G^T^, pL4C/U^T^
	**DNA G-rich Oligonucleotide Sequence**	
DNA-CCC	C-GGG-C-GGG-C-CTTCAAGTCCGGCA	DNA-CCC/G^T^, DNA-CCC/U^T^
	**G4 Ligand Bearing RNA G-rich Oligonucleotide**	
L-CCC	o-BMVC-C3-aminolinker-C-GGG-C-GGG-C-CUUCAAGUCCGGCA	L-CCC/G^T^, L-CCC/U^T^
	**Target Oligonucleotide Sequences**	
G^T^	UGCCGGACUUGAAG-UA-GGGCGGGC	
U^T^	UGCCGGACUUGAAG-UA-GGGCUGGC	
	**Control Oligonucleotide Sequences**	
DX	UGCCGGACUUGAAG/CUUCAAGUCCGGCA	
2Q	CGGGCGGGC	

p: phosphate group; a: abasic; L2: 1,2-ethanediol; L3: 1,3-propanediol; L4: 1,4-butanediol; o-BMVC: G-quadruplex binder.

**Table 2:** Thermal stability for QDH and Dss structures in potassium phosphate buffer.

QD/G^T^	60 mM K^+^	QD/U^T^	60 mM K^+^
	T_m_ (°C)		T_m_ (°C)
CCC/G^T^	69.4	CCC/U^T^	61.0
CCC-OMe/G^T^	71.7	CCC-OMe/U^T^	63.5
paa/G^T^	68.0	paa/U^T^	61.5
Aaa/G^T^	69.0	Aaa/U^T^	61.0
paC/G^T^	67.5	paC/G^T^	61.0
AaC/G^T^	68.5	AaC/G^T^	62.0
pCa/G^T^	67.4	pCa/G^T^	60.5
pL2C/G^T^	68.0	pL2C/G^T^	61.5
pL3C/G^T^	68.5	pL3C/G^T^	61.5
pL4C/G^T^	68.5	pL4C/G^T^	60.5
DNA-CCC/G^T^	61.4	DNA-CCC/U^T^	59.5

## Supplementary Materials

The following are available online at https://www.mdpi.com/article/10.3390/biom11081236/s1, Figure S1: The purity and homogeneity of o-BMVC-C3-CCC oligonucleotide verified by 15% denaturing gel electrophoresis, Figure S2: CD spectra of CCC/G^T^ and CCC/U^T^ in the presence of 50 mM KCl, 10 mM potassium phosphate and 0.1mM EDTA, pH 6.8; 25 °C, Figure S3: UV-melting profiles of CCC/G^T^ and CCC/U^T^ in the presence of 50 mM KCl, 10 mM potassium phosphate and 0.1mM EDTA, pH 6.8, Figure S4: Monitoring the increasing level of higher-order structure as a function of time. The migration of G^T^, CCC, and CCC/G^T^ molecules in the presence of 50 mM KCl by non-denaturing PAGE 20%. Gel was post-stained with NMM solution to detect G4 motifs, Figure S5: CD spectra of DNA-CCC/G^T^ and DNA-CCC/U^T^ in the presence of 50 mM KCl, 10 mM potassium phosphate and 0.1mM EDTA, pH 6.8; 25 °C, Figure S6: UV-melting curves of DNA-CCC/G^T^ hybrid in the presence of 50 mM KCl, 10 mM potassium phosphate and 0.1mM EDTA, pH 6.8, Figure S7: Comparison of the imino region of the ^1^H NMR spectra of CCC/G^T^, CCC-OMe/G^T^, paa/G^T^, Aaa/G^T^, paC/G^T^, AaC/G^T^, pCa/G^T^, pL2C/G^T^, pL3C/G^T^, pL4C/G^T^ (A) and CCC/U^T^, CCC-OMe/U^T^, paa/U^T^, Aaa/U^T^, paC/U^T^, AaC/U^T^, pCa/U^T^, pL2C/U^T^, pL3C/U^T^, pL4C/U^T^ complexes (B) at 25 °C in the presence of 150 mM NaCl, 10 mM sodium phosphate, 0.1 mM EDTA, pH 6.8, Figure S8: Schematic representation of the two possible models of the quadruplex–duplex hybrid, Figure S9: Fluorescence spectra of NMM and NMM mixed with QD/G^T^ (solid lines) and QD/U^T^ (dotted lines) molecules in a buffer containing 50 mM KCl, 10 mM potassium phosphate, pH 6.8 at 25 °C, Figure S10: Comparison of the imino region of ^1^H NMR spectra of paa/G^T^ (A), Aaa/U^T^ (B), and paa/U^T^ (C) without and with a 1:1 molar ratio of NMM in a buffer containing 50 mM KCl, 10 mM potassium phosphate, pH 6.8 at 25 °C, Figure S11: Migration of CCC/G^T^, CCC/U^T^ and chemically modified QD/G^T^ and QD/U^T^ complexes in the presence of 150 mM NaCl in non-denaturing PAGE 20%; gels were visualized by UV light (254 nm) to detect all RNAs and post-stained with NMM solution to detect G4 motifs, Table S1: Thermal stability for QDH and Dss structures in sodium phosphate buffer, Figure S12: The UV-melting profiles of QD/G^T^ at 260 and 295 nm in the presence of 50 mM KCl, 10 mM potassium phosphate, 0.1 mM EDTA, pH 6.8 (A) and 150 mM NaCl, 10 mM sodium phosphate, 0.1 mM EDTA, pH 6.8 (B), Figure S13: The UV-melting profiles of QD/U^T^ at 260 and 295 nm in the presence of 50 mM KCl, 10 mM potassium phosphate, 0.1 mM EDTA, pH 6.8 (A) and 150 mM NaCl, 10 mM sodium phosphate, 0.1 mM EDTA, pH 6.8 (B), Figure S14: Temperature dependence of the imino region of ^1^H NMR spectra for CCC/G^T^, paa/G^T^, and pL3C/G^T^ in the presence of 50 mM KCl (A) and 150 mM NaCl (B), Figure S15: Circular dichroism (CD) spectra of QDH in the presence of potassium and sodium cations, Figure S16. Circular dichroism (CD) spectra of molecules indicated in the legend in the presence of 50 mM KCl, 10 mM potassium phosphate, 0.1 mM EDTA, pH 6.8, Figure S17: o-BMVC-C3-CCC oligoribonucleotide synthesis scheme, Figure S18: The UV-melting profiles of CCC/G^T^, o-BMVC-C3-CCC/G^T^, and o-BMVC-C3-CCC/U^T^ at 260 nm. The spectra were recorded in the presence of 50 mM KCl, 10 mM potassium phosphate and 0.1 mM EDTA, pH 6.8, Figure S19: Circular dichroism (CD) spectra of o-BMVC-C3-CCC/G^T^, o-BMVC-C3-CCC/U^T^, and CCC/G^T^ at 25 °C in the presence of 50 mM KCl, 10 mM potassium phosphate and 0.1 mM EDTA, pH 6.8, Figure S20. Secondary structures of CCC/U^T^ predicted by RNAstructure, Figure S21. Putative models of bimolecular paa/U^T^ and Aaa/U^T^ hybrid.
