# Supplementary material for: Impact of a Single Nucleotide Change or Non-Nucleoside Modifications in G-Rich Region on the Quadruplex–Duplex Hybrid Formation
Source: Biomolecules. 2021 Aug 18;11(8):1236. doi: 10.3390/biom11081236 (PMC8392043; doi:10.3390/biom11081236)
Supplement: Supplementary file 1 [file biomolecules-11-01236-s001.zip › biomolecules-1326107-supplementary.pdf]

Impact of a single nucleotide change or non-nucleoside modifications in G-rich region on the quadruplex-duplex hybrid formation

Dorota Gudanis[1,&,*], Karolina Zielińska[1,&], Daniel Baranowski[1],Piotr Kozłowski[1], Ryszard Kierzek[1], Zofia Gdaniec[1]

[1] Department of Biomolecular NMR, Institute of Bioorganic Chemistry, Polish Academy of Sciences, Poznan, 61-704, Poland

&- equal first authorship

* corresponding author
Email: dgud@ibch.poznan.pl, zgdan@ibch.poznan.pl

Supplementary Informations

Figure S1: The purity and homogeneity of o-BMVC-C3-CCC oligonucleotide verified by 15% denaturing gel electrophoresis,

Figure S2: CD spectra of CCC/G^T and CCC/U^T in the presence of 50 mM KCl, 10 mM potassium phosphate and 0.1mM EDTA, pH 6.8; 25 °C,

Figure S3: UV-melting profiles of CCC/G^T and CCC/U^T in the presence of 50 mM KCl, 10 mM potassium phosphate and 0.1mM EDTA, pH 6.8,

Figure S4: Monitoring the increasing level of higher order structure as a function of time. The migration of G^T, CCC, and CCC/G^T molecules in the presence of 50 mM KCl by non-denaturing PAGE 20%. Gel was post-stained with NMM solution to detect G4 motifs,

Figure S5: CD spectra of DNA-CCC/G^T and DNA-CCC/U^T in the presence of 50 mM KCl, 10 mM potassium phosphate and 0.1mM EDTA, pH 6.8; 25 °C,

Figure S6: UV-melting curves of DNA-CCC/G^T hybrid in the presence of 50 mM KCl, 10 mM potassium phosphate and 0.1mM EDTA, pH 6.8,

Figure S7: Comparison of the imino region of the ^{1}H NMR spectra of CCC/G^T, CCC-OMe/G^T, paa/G^T, Aaa/G^T, paC/G^T, AaC/G^T, pCa/G^T, pL2C/G^T, pL3C/G^T, pL4C/G^T (A) and CCC/U^T, CCC-OMe/U^T, paa/U^T, Aaa/U^T, paC/U^T, AaC/U^T, pCa/U^T, pL2C/U^T, pL3C/U^T, pL4C/U^T complexes (B) at 25 °C in the presence of 150 mM NaCl, 10 mM sodium phosphate, 0.1 mM EDTA, pH 6.8,

Figure S8: Schematic representation of the two possible models of the quadruplex–duplex hybrid,

Figure S9: Fluorescence spectra of NMM and NMM mixed with QD/G^T (solid lines) and QD/U^T (dotted lines) molecules in a buffer containing 50 mM KCl, 10 mM potassium phosphate, pH 6.8 at 25 °C,

Figure S10: Comparison of the imino region of ^{1}H NMR spectra of paa/G^T (A), Aaa/U^T (B), and paa/U^T (C) without and with a 1:1 molar ratio of NMM in a buffer containing 50 mM KCl, 10 mM potassium phosphate, pH 6.8 at 25 °C,

Figure S11: Migration of CCC/G^T, CCC/U^T and chemically modified QD/G^T and QD/U^T complexes in the presence of 150 mM NaCl in non-denaturing PAGE 20%; gels were visualized by UV light (254 nm) to detect all RNAs and post-stained with NMM solution to detect G4 motifs,

Table S1: Thermal stability for QDH and Dss structures in sodium phosphate buffer,

Figure S12: The UV-melting profiles of QD/G^T at 260 and 295 nm in the presence of 50 mM KCl, 10 mM potassium phosphate, 0.1 mM EDTA, pH 6.8 (A) and 150 mM NaCl, 10 mM sodium phosphate, 0.1 mM EDTA, pH 6.8 (B),

Figure S13: The UV-melting profiles of QD/U^T at 260 and 295 nm in the presence of 50 mM KCl, 10 mM potassium phosphate, 0.1 mM EDTA, pH 6.8 (A) and 150 mM NaCl, 10 mM sodium phosphate, 0.1 mM EDTA, pH 6.8 (B),

Figure S14: Temperature dependence of the imino region of ^{1}H NMR spectra for CCC/G^T, paa/G^T, and pL3C/G^T in the presence of 50 mM KCl (A) and 150 mM NaCl(B),

Figure S15: Circular dichroism (CD) spectra of QDH in the presence of potassium and sodium cations,

Figure S16. Circular dichroism (CD) spectra of molecules indicated in the legend in the presence of 50 mM KCl, 10 mM potassium phosphate, 0.1 mM EDTA, pH 6.8,

Figure S17: o-BMVC-C3-CCC oligoribonucleotide synthesis scheme,

Figure S18: The UV-melting profiles of CCC/G^T, o-BMVC-C3-CCC/G^T, and o-BMVC-C3-CCC/U^T at 260 nm. The spectra were recorded in the presence of 50 mM KCl, 10 mM potassium phosphate and 0.1 mM EDTA, pH 6.8,

Figure S19: Circular dichroism (CD) spectra of o-BMVC-C3-CCC/G^T, o-BMVC-C3-CCC/U^T, and CCC/G^T at 25 °C in the presence of 50 mM KCl, 10 mM potassium phosphate and 0.1 mM EDTA, pH 6.8,

Figure S20: Secondary structures of CCC/U^T predicted by RNAstructure,

Figure S21. Putative models of bimolecular paa/U^T and Aaa/U^T hybrid.

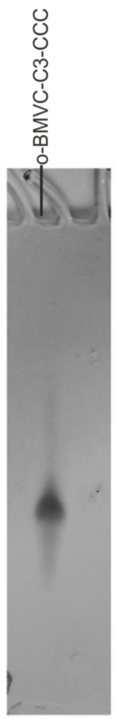

Figure S1: The purity and homogeneity of o-BMVC-C3-CCC oligonucleotide verified by 15% denaturing gel electrophoresis.

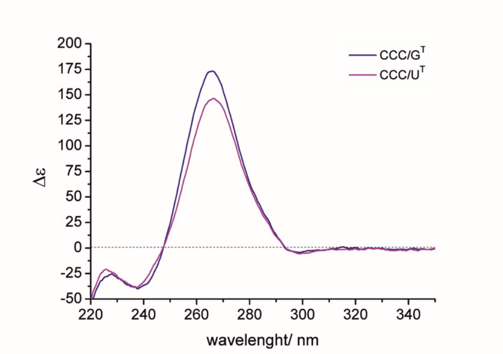

Figure S2: CD spectra of CCC/G^T and CCC/U^T in the presence of 50 mM KCl, 10 mM potassium phosphate and 0.1mM EDTA, pH 6.8; 25 °C.

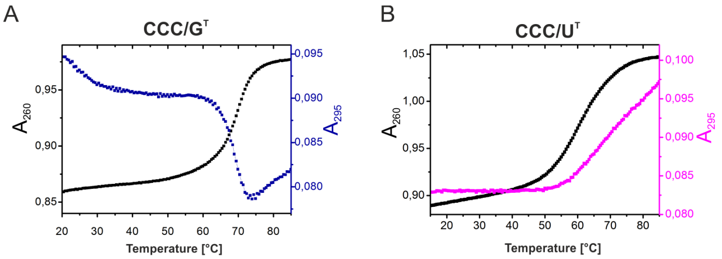

Figure S3: UV-melting profiles of CCC/G^T and CCC/U^T in the presence of 50 mM KCl, 10 mM potassium phosphate and 0.1mM EDTA, pH 6.8.

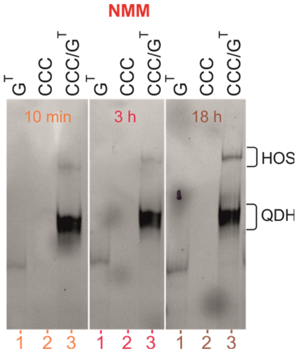

Figure S4: Monitoring the increasing level of higher order structure as a function of time. The migration of G^T, CCC, and CCC/G^T molecules in the presence of 50 mM KCl by non-denaturing PAGE 20%. Gel was post-stained with NMM solution to detect G4 motifs.

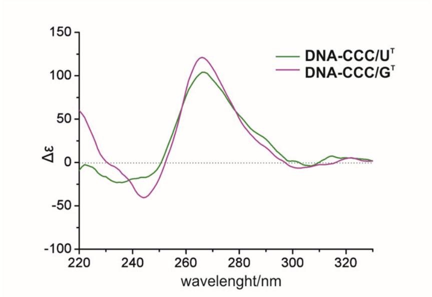

Figure S5: CD spectra of DNA-CCC/G^T and DNA-CCC/U^T in the presence of 50 mM KCl, 10 mM potassium phosphate and 0.1mM EDTA, pH 6.8; 25 °C.

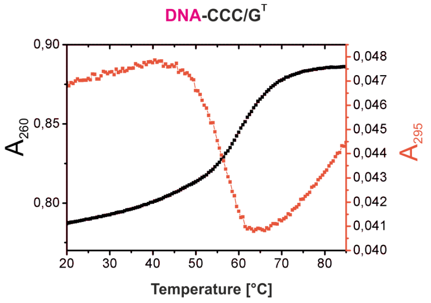

Figure S6: UV-melting curves of DNA-CCC/G^T hybrid in the presence of 50 mM KCl, 10 mM potassium phosphate and 0.1mM EDTA, pH 6.8.

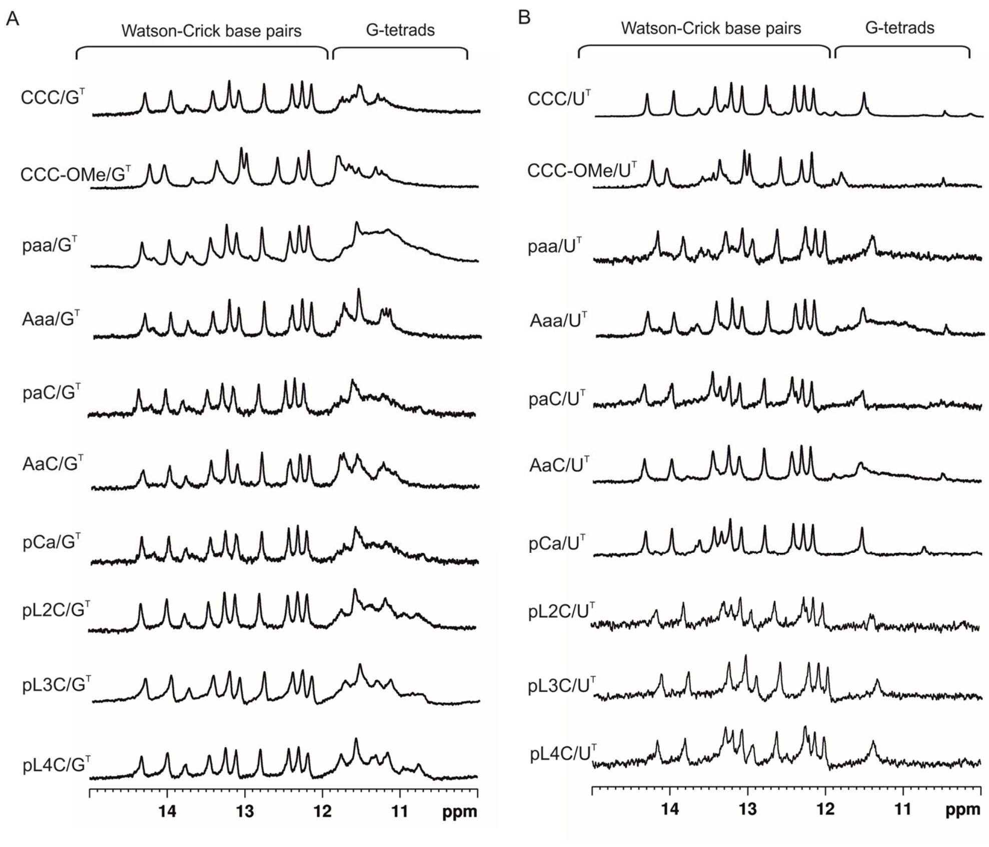

Figure S7: Comparison of the imino region of the ^1H NMR spectra of CCC/G^T, CCC-OMe/G^T, paa/G^T, Aaa/G^T, paC/G^T, AaC/G^T, pCa/G^T, pL2C/G^T, pL3C/G^T, pL4C/G^T (A) and CCC/U^T, CCC-OMe/U^T, paa/U^T, Aaa/U^T, paC/U^T, AaC/U^T, pCa/U^T, pL2C/U^T, pL3C/U^T, pL4C/U^T complexes (B) at 25 °C in the presence of 150 mM NaCl, 10 mM sodium phosphate, 0.1 mM EDTA, pH 6.8.

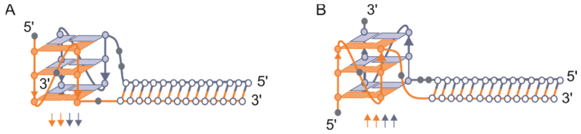

Figure S8: Schematic representation of the two possible models of the quadruplex–duplex hybrid.

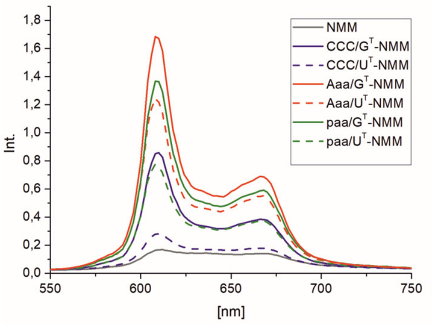

Figure S9: Fluorescence spectra of NMM and NMM mixed with QD/G^T (solid lines) and QD/U^T (dotted lines) molecules in a buffer containing 50 mM KCl, 10 mM potassium phosphate, pH 6.8 at 25 °C.

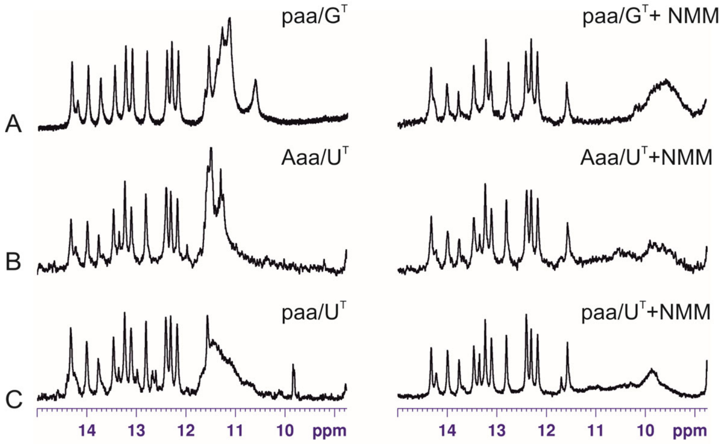

Figure S10: Comparison of the imino region of ^{1}H NMR spectra of paa/G^T (A), Aaa/U^T (B), and paa/U^T (C) without and with a 1:1 molar ratio of NMM in a buffer containing 50 mM KCl, 10 mM potassium phosphate, pH 6.8 at 25 °C.

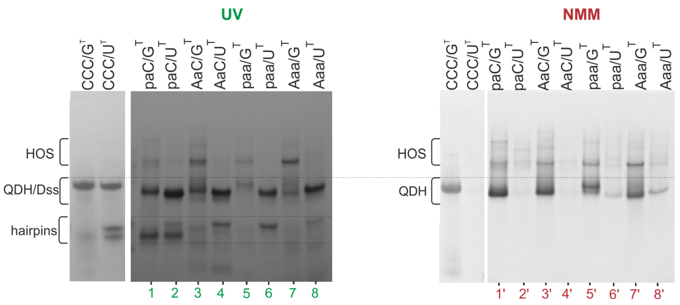

Figure S11: Migration of CCC/G^T, CCC/U^T and chemically modified QD/G^T and QD/U^T complexes in the presence of 150 mM NaCl in non-denaturing PAGE 20%; gels were visualized by UV light (254 nm) to detect all RNAs and post-stained with NMM solution to detect G4 motifs.

Table S1: Thermal stability for QDH and Dss structures in sodium phosphate buffer.

**QD/G^T**	**160 mM Na^+**	**QD/U^T**	**160 mM Na^+**
	**T_m [°C]**		**T_m [°C]**
**CCC/G^T**	71.0	**CCC/U^T**	70.5
**CCC-OMe/G^T**	75.6	**CCC-OMe/U^T**	74.5
**paa/G^T**	69.6	**paa/U^T**	70.0
**Aaa/G^T**	69.5	**Aaa/U^T**	69.0
**paC/G^T**	69.8	**paC/G^T**	70.0
**AaC/G^T**	69.5	**AaC/G^T**	70.0
**pCa/G^T**	69.5	**pCa/G^T**	69.0
**pL2C/G^T**	69.6	**pL2C/G^T**	70.7
**pL3C/G^T**	70.7	**pL3C/G^T**	69.0
**pL4C/G^T**	69.5	**pL4C/G^T**	67.2

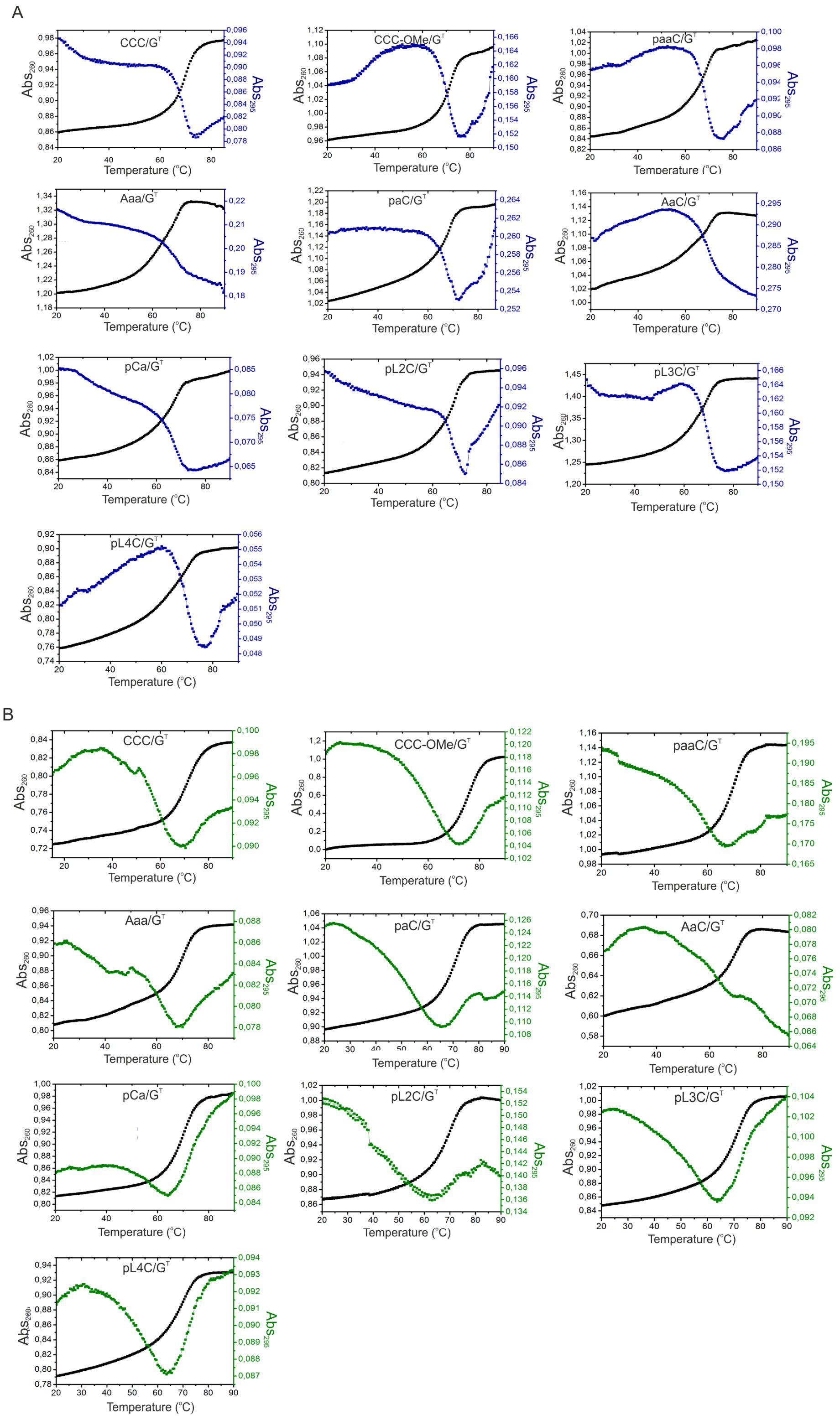

Figure S12: The UV-melting profiles of QD/G^T at 260 and 295 nm in the presence of 50 mM KCl, 10 mM potassium phosphate, 0.1 mM EDTA, pH 6.8 (A) and 150 mM NaCl, 10 mM sodium phosphate, 0.1 mM EDTA, pH 6.8 (B).

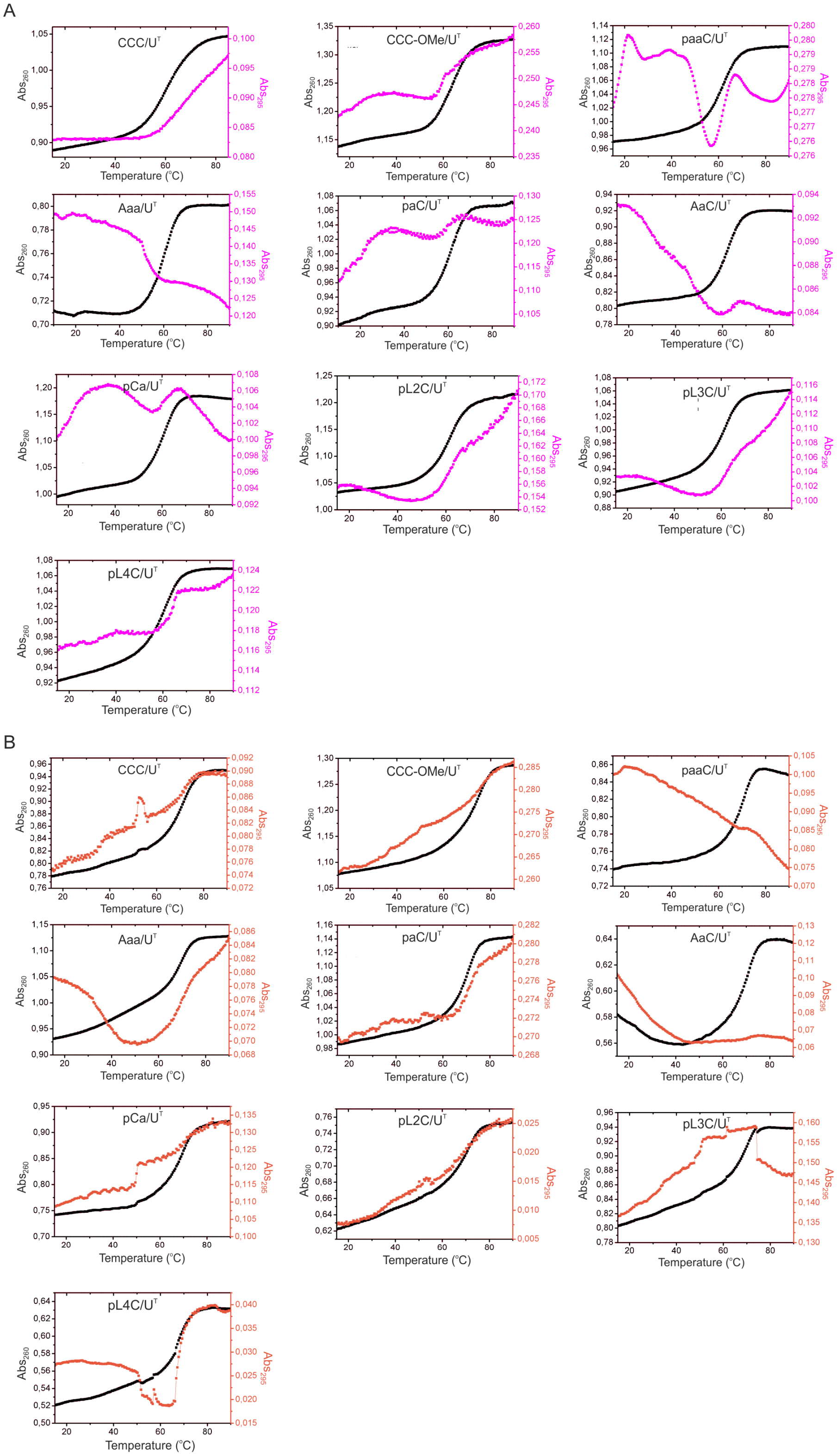

Figure S13: The UV-melting profiles of QD/U^T at 260 and 295 nm in the presence of 50 mM KCl, 10 mM potassium phosphate, 0.1 mM EDTA, pH 6.8 (A) and 150 mM NaCl, 10 mM sodium phosphate, 0.1 mM EDTA, pH 6.8 (B).

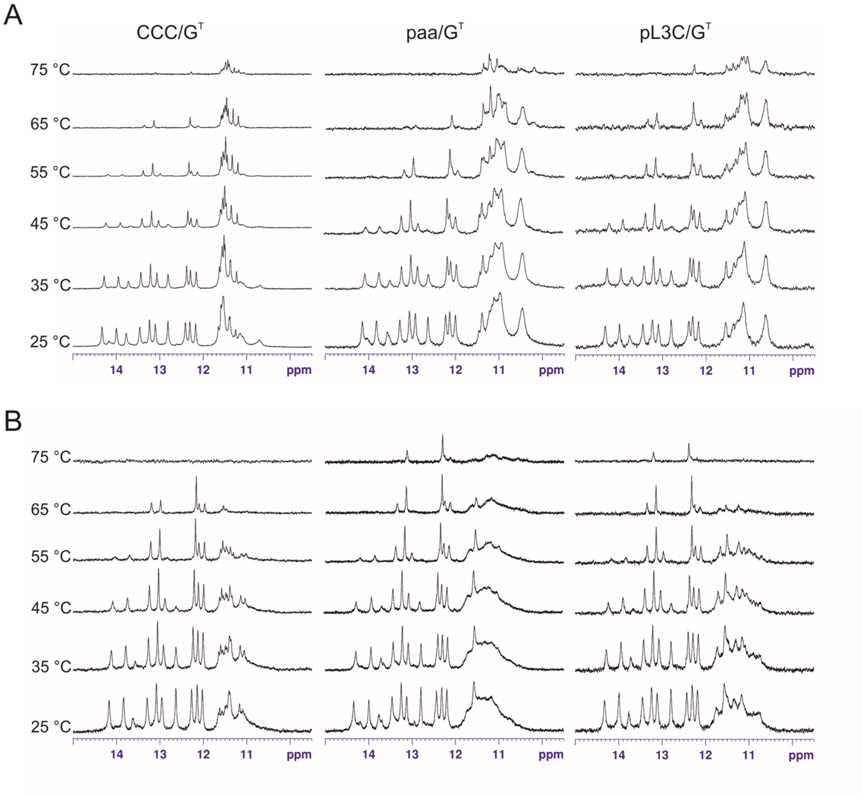

Figure S14: Temperature dependence of the imino region of ^{1}H NMR spectra for CCC/G^T, paa/G^T, and pL3C/G^T in the presence of 50 mM KCl (A) and 150 mM NaCl (B).

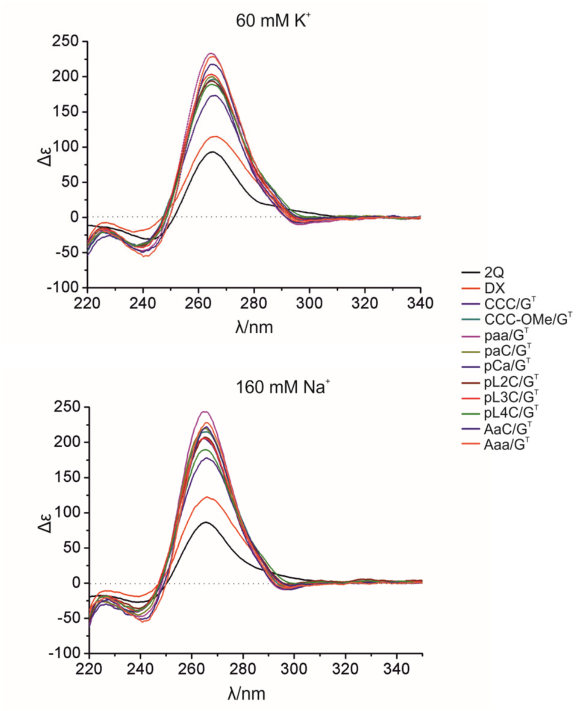

Figure S15: Circular dichroism (CD) spectra of QDH in the presence of potassium and sodium cations.

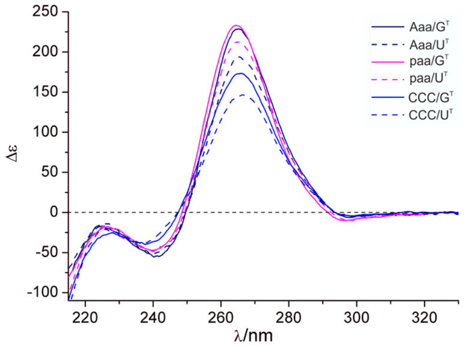

Figure S16. Circular dichroism (CD) spectra of molecules indicated in the legend in the presence of 50 mM KCl, 10 mM potassium phosphate, 0.1 mM EDTA, pH 6.8.

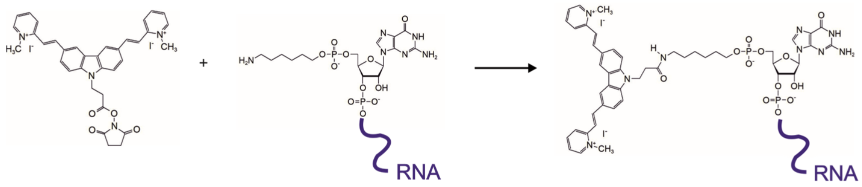

Figure S17: o-BMVC-C3-CCC oligoribonucleotide synthesis scheme.

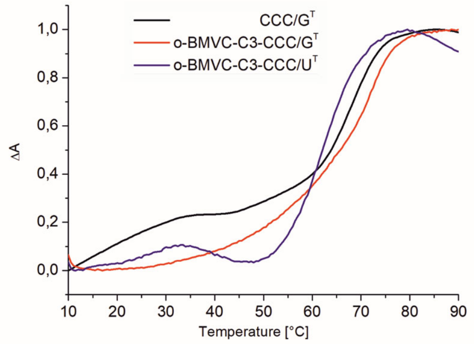

Figure S18: The UV-melting profiles of CCC/G^T, o-BMVC-C3-CCC/G^T, and o-BMVC-C3-CCC/U^T at 260 nm. The spectra were recorded in the presence of 50 mM KCl, 10 mM potassium phosphate and 0.1 mM EDTA, pH 6.8.

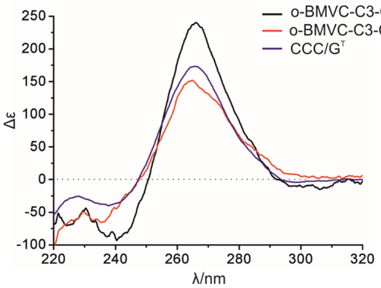

Figure S19: Circular dichroism (CD) spectra of o-BMVC-C3-CCC/G^T, o-BMVC-C3-CCC/U^T, and CCC/G^T at 25 °C in the presence of 50 mM KCl, 10 mM potassium phosphate and 0.1 mM EDTA, pH 6.8.

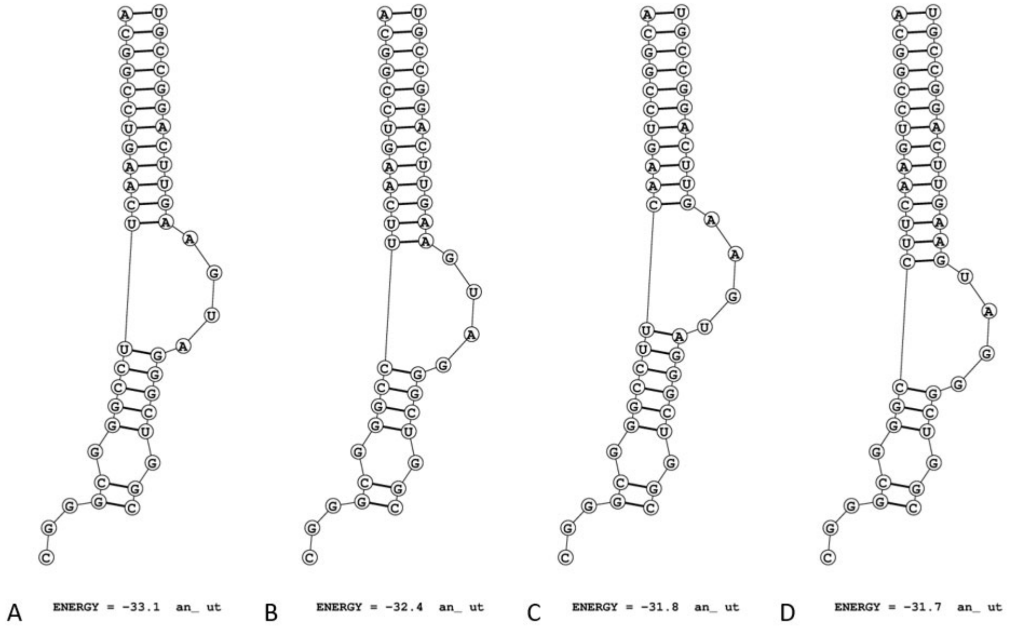

Figure S20: Secondary structures of CCC/U^T predicted by RNAstructure.

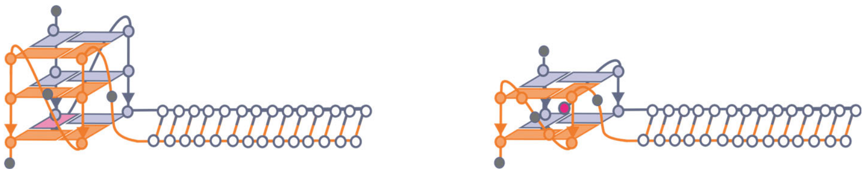

Figure S21. Putative models of bimolecular paa/U^T and Aaa/U^T hybrid.
